# Extracellular vesicles from organoid‐derived human retinal progenitor cells prevent lipid overload‐induced retinal pigment epithelium injury by regulating fatty acid metabolism

**DOI:** 10.1002/jev2.12401

**Published:** 2023-12-27

**Authors:** Hui Gao, Yuxiao Zeng, Xiaona Huang, Luodan A, Qingle Liang, Jing Xie, Xi Lin, Jing Gong, Xiaotang Fan, Ting Zou, Haiwei Xu

**Affiliations:** ^1^ Southwest Eye Hospital, Southwest Hospital Third Military Medical University (Army Medical University) Chongqing China; ^2^ Key Lab of Visual Damage and Regeneration & Restoration of Chongqing Chongqing China; ^3^ Department of Clinical Laboratory Medicine, First Affiliated Hospital Third Military Medical University (Army Medical University) Chongqing China; ^4^ Key Laboratory of Biorheological Science and Technology, Ministry of Education, College of Bioengineering Chongqing University Chongqing China; ^5^ Department of Military Cognitive Psychology, School of Psychology Third Military Medical University (Army Medical University) Chongqing China; ^6^ Department of Ophthalmology The Second Affiliated Hospital of Chongqing Medical University Chongqing China

**Keywords:** extracellular vesicles, lipid metabolism, retinal organoids, retinal pigment epithelium cells, retinal progenitor cells

## Abstract

Retinal degeneration (RD), a group of diseases leading to irreversible vision loss, is characterised by retinal pigment epithelium (RPE) or retinal neuron damage and loss. With fewer risks of immune rejection and tumorigenesis, stem cell‐secreted extracellular vesicles (EVs) offer a new cell‐free therapeutic paradigm for RD, which remains to be investigated. Human retinal organoid‐derived retinal progenitor cells (hERO‐RPCs) are an easily accessible and advanced cell source for RD treatment. However, hERO‐RPCs‐derived EVs require further characterisation. Here, we compared the characteristics of EVs from hERO‐RPCs (hRPC‐EVs) with those of human embryonic stem cell (hESC)‐derived EVs (hESC‐EVs) as controls. Based on in‐depth proteomic analysis, we revealed remarkable differences between hRPC‐EVs and hESC‐EVs. A comparison between EVs and their respective cells of origin demonstrated that the protein loading of hRPC‐EVs was more selective than that of hESC‐EVs. In particular, hESC‐EVs were enriched with proteins related to angiogenesis and cell cycle, whereas hRPC‐EVs were enriched with proteins associated with immune modulation and retinal development. More importantly, compared with that of hESC‐EVs, hRPC‐EVs exhibited a lower correlation with cell proliferation and a unique capacity to regulate lipid metabolism. It was further confirmed that hRPC‐EVs potentially eliminated lipid deposits, inhibited lipotoxicity and oxidative stress, and enhanced phagocytosis and survival of oleic acid‐treated ARPE‐19 cells. Mechanistically, hRPC‐EVs are integrated into the mitochondrial network of oleic acid‐treated ARPE‐19 cells, and increased the level of mitochondrial fatty acid β‐oxidation‐related proteins. Thus, organoid‐derived hRPC‐EVs represent a promising source of cell‐free therapy for RD, especially for blinding diseases related to abnormal lipid metabolism in RPE cells.

## 1 INTRODUCTION

Retinal degeneration (RD), a group of severe diseases causing irreversible vision loss, such as age‐related macular degeneration (AMD) and retinitis pigmentosa (RP), leads to complete loss of visual function at the late development stage (Hartong et al., 2006; Mitchell et al., 2018; Stein et al., 2021). Loss of retinal pigment epithelium (RPE) or retinal neurons is a similar and core pathological feature in the pathophysiology of RD. Stem cell‐based therapy is considered a promising strategy to treat RD, replenishing lost retinal neurons and RPE (Gasparini et al., 2019; Singh et al., 2020) or protecting remaining cells through the bystander effect (Bian et al., 2020). Several clinical trials have shown the safety and potential effectiveness of stem cells in treating RD (Stern et al., 2018). Therefore, exploring optimised cell sources and products is of great significance.

Embryonic stem cells (ESCs) or induced pluripotent stem cells (iPSCs), characterised by pluripotency and unlimited self‐renewal, are potentially ideal sources for treating RD. However, concerns regarding the safety of ESC‐derived cells remain to be addressed. ESCs and iPSCs rapidly acquire oncogenic mutations during expansion, eventually leading to tumorigenesis (Merkle et al., 2017). Therefore, these cells are usually differentiated into retinal cells before transplantation to reduce the risk of tumorigenesis. It was shown that human embryonic stem cell (hESC)‐derived RPE cells grafted in the subretinal space of patients with AMD or Stargardt's disease (STD) were safe and effective in our early‐phase clinical trials (Li et al., 2021; Liu et al., 2018). Notably, adverse proliferation and immune rejection are also concerns when ESC‐ or iPSC‐differentiated cells, especially retinal progenitor cells (RPCs) or photoreceptor precursors, are used to treat RD (Cui et al., 2013; Stern et al., 2018). The emergence of retinal organoid technology offers a more appropriate cell source, as stem cell differentiation more closely resembles a naïve developmental process for purifying differentiated cells, ensuring optimum safety and potency before transplantation (Eiraku et al., 2011; Gonzalez‐Cordero et al., 2013). Moreover, compared with retinal progenitors/precursors cultured in traditional 2D culture, those isolated from retinal organoids mature into photoreceptor outer segments and integrate into the retina more efficiently (Gonzalez‐Cordero et al., 2013). Therefore, organoid‐derived retinal progenitors/precursors are optimal sources for retinal transplantation. Nevertheless, ESC‐ or iPSC‐derived RPCs or precursors have not yet been used in clinical trials because the possibility of mixing undifferentiated cells during the differentiation process cannot be completely ruled out. In addition to safety concerns, poor survival and immunological rejection are inevitable and limit the clinical translation of stem cell therapy (Singh et al., 2020). Cell‐free therapy mediated by extracellular vesicles (EVs) may be a safer and promising alternative for treating RD.

EVs typically contain exosomes, microvesicles and apoptotic bodies and are secreted from all types of cells, including stem cells. EVs play an essential role in cell communication and serve as biomarkers. As critical mediators, EVs are involved in material transfer during stem cell therapy; however, this requires further investigation (Bian et al., 2020; Zou et al., 2023). In addition, as a cell‐free therapeutic strategy, EV transplantation is considered to exert therapeutic effects while avoiding the side effects of cell transplantation (Deng et al., 2021). Stem cell‐secreted EVs could yield multiple effects by delivering functional mRNA, miRNA, lipids and proteins, thereby promoting proliferation, differentiation, neuroprotection and immune regulation, as well as maintaining homeostasis of the microenvironment (Didiot et al., 2016; Haney et al., 2013; Kojima et al., 2018; Yang et al., 2017). Recently, EV transplantation was shown to play a positive role in several retinal diseases (Mead & Tomarev, 2020). Previously, we also showed that neural progenitor cell‐derived EVs were specifically internalised by microglia and suppressed microglial activation via miRNA, thus delaying photoreceptor death during RD (Bian et al., 2020). However, EVs of different cells are distinctly loaded with diverse proteins that might determine their distinct functions in retinal repair and regeneration (Klingeborn et al., 2017; Zhou et al., 2018). Therefore, exploring the protein cargo of stem cell‐derived EVs and screening for optimal therapeutic EVs could significantly contribute to the development of EV‐mediated therapies for RD.

EVs from ESCs or iPSCs potentially play a critical role in the maintenance of stem cell pluripotency and somatic cell de‐differentiation and could possibly provide protective effects in injured organs (Khan et al., 2015; Peng et al., 2018; Zhou et al., 2016). In addition, ESC‐ or iPSC‐derived cells secrete functional EV cargoes with better properties than those of EVs secreted from adult cells, such as iPSC‐derived mesenchymal stem cells (MSCs) (Kim et al., 2018). However, pluripotency‐related proteins that are present in the EV cargo of undifferentiated pluripotent stem cells raise safety concerns regarding their application (Peng et al., 2018; Zhou et al., 2016). We previously reported that RPCs from human ESC‐derived retinal organoids (hEROs) restored visual function in RD animals by replacing lost photoreceptors and transferring cytoplasmic material into host photoreceptors without tumorigenesis or abnormal proliferation (Zou et al., 2019). As described above, retinal organoid‐derived RPCs have shown improved safety and therapeutic potential (Gonzalez‐Cordero et al., 2013). Whether EVs secreted from differentiated hERO‐RPCs (herein referred to as hRPC‐EVs) are an ideal source for cell‐free therapy of RD remains to be determined.

In this study, we successfully harvested EVs derived from hESCs and hERO‐RPCs (hESC‐EVs and hRPC‐EVs, respectively). Our 4D label‐free proteomic analysis of hESCs, hESC‐EVs, hERO‐RPCs (hRPCs) and hRPC‐EVs showed remarkable differences in protein content among the four groups. Compared to their cells of origin, hESC‐EVs were enriched with angiogenesis‐related proteins, whereas hRPC‐EVs were enriched with proteins associated with immune modulation and retinal development. Functional comparisons between hESC‐EVs and hRPC‐EVs revealed the ability of hESC‐EVs to promote cell proliferation, whereas hRPC‐EVs showed a remarkable ability to regulate lipid metabolism. Finally, we confirmed that hRPC‐EVs could protect RPE cells from lipid overload‐induced cytotoxicity by regulating fatty acid metabolism, shedding light on the treatment of lipid dysregulation‐related RD.

## 2 MATERIALS AND METHODS

### 2.1 Induction of human embryonic stem cell‐derived retinal organoids

In vitro experiments using human cell line were approved by the Committee on Ethics of the Southwest Hospital, Army Medical University (Chongqing, China). hESC line (Q‐CTS‐hESC‐2) used in the present study was a gift from the Chinese Academy of Sciences (Gu et al., 2017). The hESCs were cultured in Essential 8 Culture Medium (A1517001; Gibco, USA) without feeders. When the hESCs reached 80% confluence, they were dissociated into single‐cell suspensions using TrypLE Express (12604013; Gibco) containing 0.05 mg/mL DNase I (03724778103; Roche, Switzerland) and 20 mM Y‐27632 (SCM075; Merck, Germany) (Kuwahara et al., 2015; Zou et al., 2019). The medium for hERO induction consisted of 45% F12‐Glutamax (31765035, Gibco), 45% IMDM (12440053; Gibco), 450 µM monothioglycerol (M6145; Sigma‐Aldrich, USA), 20 µM Y‐27632, and 1% chemically defined lipid concentrate (11905031; Gibco) supplemented with 10% knockout serum replacement (10828028; Gibco). Initially, the cell suspension (1.2 × 10^4^ cells, 100 µL) was added into low‐cell‐attachment 96‐well plates with each well having a V‐bottom (MS‐9096; Sumitomo Bakelite, Japan) and cultured for 6 days. Next, the medium was replaced with fresh hERO induction medium containing 1.5 nM bone morphogenetic protein 4 (BMP4: 120‐05ET; PeproTech, USA), and half of this medium was replaced every 72 h. On day 18, hEROs were transferred to NEST low‐cell‐attachment culture dishes in Dulbecco's modified Eagle's medium (DMEM)/F12‐Glutamax supplemented with 10% fetal bovine serum (FBS; 26010074; Gibco), 1% N2 supplement (17502048; Gibco), 0.1 mM taurine (T8691; Sigma‐Aldrich) and 0.5 µM retinoic acid (R2625; Sigma‐Aldrich). The hEROs and all cultured cells were incubated in a standard incubator (37°C, 5% CO_2_, saturated humidity).

### 2.2 Fluorescence‐activated cell sorting and hRPC culture

As previously described (Zou et al., 2019), hEROs were dissociated into single‐cell suspensions on day 30 using TrypLE Express. Cell suspensions (1 × 10^6^/100 µL) were incubated with FITC‐conjugated anti‐human SSEA‐4 antibody (560126; BD Biosciences, USA) and κ isotype control antibody (555578; BD Biosciences) for 30 min at 4°C. hRPCs were separated through fluorescence‐activated cell sorting (FACS) on a BD FACS Aria II (BD Biosciences) and were cultured in UltraCULTURE medium (12‐725F; Lonza, Switzerland) containing 20 ng/mL human basic fibroblast growth factor (100‐18B; PeproTech), 10 ng/mL human epidermal growth factor (GMP100‐15; PeproTech), 1% N2, 20 µM Y‐27632, 2% B27 (17504044, Gibco) and 1% Glutamax supplement (35050061, Gibco) for 24 h at 37°C in a standard incubator. The medium was replaced after 24 h, and the hRPCs were passaged every 72 h.

### 2.3 Sample preparation and immunofluorescence staining

To identify hEROs and cultured cells, immunofluorescence staining was performed as described in our previous study (Zou et al., 2019). Randomly selected hEROs were fixed in 4% paraformaldehyde (PFA) at 4°C for 30 min before dehydration in 30% sucrose solution at 4°C overnight. Then, hEROs were embedded with optimal cutting temperature (4583; Sakura FineTek, USA) and were frozen at −80°C. Specimens were cut into 10‐µm thick sections using a cryostat (CM1900UV; Leica, Germany). Sections were mounted on glass slides before being stored at −20°C in a freezer until immunofluorescence staining. For cell preparation, hESCs and hRPCs seeded on slides were fixed with 4% PFA at room temperature for 15 min. For immunofluorescence staining, slides or sections were washed thrice with 0.01 M phosphate‐buffered saline (PBS). After permeabilisation in 0.5% Triton X‐100 for 10 min, the slides or sections were blocked in 0.3% Triton X‐100 containing 3% bovine serum albumin for 30 min at room temperature. Afterward, the slides or sections were incubated with primary antibodies at 4°C overnight in a humidified chamber. The following primary antibodies and dilutions were used in this study: mouse anti‐SSEA4 (sc‐59368, 1:100; Santa Cruz Biotechnology, USA), mouse anti‐CHX10 (sc‐374151, 1:300; Santa Cruz Biotechnology), rabbit anti‐CHX10 (HPA003436 1:500; Sigma‐Aldrich), mouse anti‐RAX (sc‐271889, 1:500; Santa Cruz Biotechnology), mouse anti‐OCT4 (sc‐5279, 1:100; Santa Cruz Biotechnology), mouse anti‐NANOG (sc‐374103, 1:100; Santa Cruz Biotechnology), rabbit anti‐PAX6 (ab5790, 1:500; Abcam, UK) and mouse anti‐HuC/D (A21272, 1:200; Invitrogen, USA). The next day, sections were incubated with the following secondary antibodies respectively: goat anti‐rabbit IgG Alexa‐Fluor‐568 (A11011, 1:500; Life Technologies, USA), goat anti‐mouse IgG Alexa‐Fluor‐568 (A11031, 1:500; Life Technologies), goat anti‐mouse lgG Alexa‐Fluor‐488 (A11001, 1:500; Life Technologies) and goat anti‐rabbit IgG Alexa‐Fluor‐488 (A11008, 1:500; Life Technologies) for 60 min at 37°C before washing with PBS. After nuclear counterstaining with DAPI (C1005；Beyotime Biotechnology, China), slides or sections were photographed under a confocal laser scanning microscope (LSM 880; Zeiss, Germany).

### 2.4 Extracellular vesicle isolation

For hRPC‐EV isolation, 1.5 × 10^7^ cells were seeded into two T75 culture flasks (10 mL culture medium per T75 culture flask) and cultured for 72 h. When the cell confluence reached 80%, the medium was replaced with fresh medium. The conditioned medium was collected after culturing for 48 h to isolate EVs. For hESC‐EV isolation, 6 × 10^6^ cells were seeded into two T75 culture flasks (10 mL culture medium per T75 culture flask) and cultured for 72 h. When the cell confluence reached 80%, the medium was replaced with fresh medium. After 24 h of culture, the conditioned medium was collected for hESC‐EV isolation. As previously described (Bian et al., 2020; Chen et al., 2021; Ikeda et al., 2021), conditioned medium from hESCs and hRPCs was collected and centrifuged at 1500 rpm for 15 min and at 2500 rpm for 15 min (both at 4°C), respectively, to remove cellular debris. Then, the conditioned medium was ultracentrifuged at 100,000 × *g* for 70 min using a Beckman OPTIMA XPN‐100 (Beckman Coulter) at 4°C. The pellet was resuspended in PBS and centrifuged at 100,000 × *g* for 70 min at 4°C. Afterward, EVs were resuspended in PBS and stored at −80°C.

### 2.5 Transmission electron microscopy

As previously reported (Bian et al., 2020; Huang et al., 2023; Hugendieck et al., 2023), EVs were placed on Formvar‐coated 400‐mesh copper grids for 20 min until adsorption at room temperature. EVs were fixed in a solution containing 2% PFA, 0.05 M phosphate and 2% glutaraldehyde for 2 min. The grids were washed thrice with PBS to remove excess mixture and negatively stained with 1% phosphotungstic acid. The EV ultrastructure was visualised using transmission electron microscopy (TEM; JEM‐1400PLUS, JEOL, Japan).

### 2.6 Nanoparticle tracking analysis

As previously reported (Liao et al., 2019; Visan et al., 2022), the concentrated EV solution was diluted 1000 times and injected into a ZetaView instrument (Particle Metrix, Germany) using a sterile injection syringe. The EV solution was analysed by scanning at 11 positions. Parameters were as follows: sensitivity, 80; shutter, 100; frame rate, 30; focus, auto‐alignment; laser wavelength, 488 nm; temperature, 25°C; pH, 7.4. Acquired videos were analysed for the concentration and size distribution of EVs using ZetaView Software (version 8.05.12 SP2) with the following parameters: max area, 1000; min area, 10; min bright, 30; laser wavelength, 488 nm.

### 2.7 Western blotting

Cells or EVs were lysed with lysis buffer comprising 10% phenylmethylsulfonyl fluoride (ST506; Beyotime Biotechnology, China) and 90% RIPA (R0010; Solarbio, China) to extract proteins, and the protein concentration was measured using the BCA protein quantitation kit (P0012; Beyotime Biotechnology). Samples were loaded and separated on a 10% SDS‐PAGE gel. A loading buffer containing DTT (R0861; Thermo Fisher Scientific, USA) was used. The proteins were then electroblotted onto a PVDF membrane at 80 V for stacking and 100 V for separation. Next, the samples were blocked in TBST buffer supplemented with 5% skimmed milk for 30 min and incubated with rabbit anti‐CD81 (ExoAB Antibody Kit, 220211‐008; System Biosciences, USA), rabbit anti‐CD63 (ExoAB Antibody Kit, 220211‐009; System Biosciences), rabbit anti‐FLOT1 (A3023; ABclonal, China), mouse anti‐GM130 (M05865‐2; Boster, USA), rabbit anti‐CRAT (A6365; ABclonal) and rabbit anti‐HADH (A1076; ABclonal) antibodies overnight at 4°C. The following day, the membranes were incubated with a goat anti‐rabbit horseradish peroxidase (HRP) secondary antibody (ExoAB Antibody Kit, 220305‐001; System Biosciences) and goat anti‐mouse HRP secondary antibody (BA1050; Boster) for 2 h at ambient temperature. Primary antibodies were used at a 1:1000 dilution and secondary antibodies at a 1:5000 dilution. After detection with Pierce ECL Western Blotting Substrate (32106; Thermo Fisher Scientific), proteins were scanned with a Bio‐Rad exposure system (Bio‐Rad, USA).

### 2.8 Proteomic analysis

For 4D label‐free proteomic analysis (Meier et al., 2018), three biological replicates were prepared for each group. Sample lysis and protein extraction were performed using SDT buffer (4% SDS, 100 mM Tris‐HCl, 1 mM DTT, pH 7.6). Thereafter, proteins were digested using trypsin (Gibco) according to the filter‐aided sample preparation procedure proposed by Matthias Mann (Wiśniewski et al., 2009). Digested peptides in each sample were desalted on C18 cartridges with 7 mm inner diameter and 3 mL volume (Empore SPE Cartridges C18 (standard density), Sigma‐Aldrich), concentrated via vacuum centrifugation, and resuspended in 40 µL of 0.1% (v/v) formic acid (Sigma‐Aldrich) (Wang et al., 2023). UV light spectral density was used to measure the peptide content at 280 nm. Liquid chromatography‐tandem mass spectrometry (LC‐MS/MS) was conducted using a timsTOF Pro mass spectrometer (Bruker, USA) coupled to a nanoelute (Bruker) (Adhikari et al., 2020). The peptides (400 ng for each sample) were loaded onto a reversed‐phase trap column connected to the C18 reversed‐phase analytical column (Thermo Scientific Easy Column; 10 cm long, 75 µm inner diameter, 3 µm resin) in 0.1% formic acid. The peptides were then separated using a linear gradient of buffer containing 0.1% formic acid and 84% acetonitrile at a flow rate of 300 nL/min (IntelliFlow Technology, London, UK). The mass spectrometer was operated in the positive ion mode for the detection and recording of ion mobility MS spectra (mass range, m/z 100−1700; 10 cycles of PASEF MS/MS; threshold, 2500; target intensity, 1.5 k).

Identification and quantitation of protein expression for each cell sample were performed using MaxQuant 1.5.3.17 from the raw MS data, which were used for bioinformatic analysis (Prianichnikov et al., 2020). Proteins and peptides with a false discovery rate (FDR) <0.01 were considered of high confidence and used for downstream analysis. Protein level was normalised to the LFQ intensity for inter‐group comparisons.

### 2.9 Bioinformatic analysis

Andromeda analysis was used to estimate the quality of the MS2 spectrum (Cox et al., 2011). Proteins with a fold change >2 or < 0.5 and *p*‐value <0.05 between the two groups were recognised as differentially expressed proteins (DEPs). Gene Ontology (GO) and Kyoto Encyclopedia of Genes and Genomes (KEGG) pathway enrichment analyses were conducted using the cluster Profiler 4.0 package in R (Yu et al., 2012). Data were plotted and visualised using the ggplot2 package. Protein–protein interactions (PPIs) were performed on the website (https://cn.string‐db.org/), and pathway–pathway interactions were determined using Cytoscape_3.5.1 (Shannon et al., 2003). GSEA v4.3.0 (https://www.gsea‐msigdb.org) was used for Gene Set Enrichment Analysis (GSEA) based on protein expression (Saha et al., 2022; Subramanian et al., 2005). Simple sample GSEA was performed using GSVA and GSEABase packages.

### 2.10 Oleic acid and extracellular vesicle treatment of ARPE‐19 cells

The human RPE cell line, ARPE‐19 that was purchased from Procell Life Science&Technology Co.,Ltd (CL‐0026; China), was cultured in DMEM/Ham's F‐12 containing 10% FBS and 100 U/mL penicillin in a humidified incubator with a 5% CO_2_ atmosphere at 37°C. After reaching 80% confluence, ARPE‐19 cells were dissociated using 0.25% trypsin for subsequent experiments. Oleic acid (HY‐N1446; MedChemExpress, China) was dissolved in DMSO at 1000 mM and stored at −80°C. For working medium preparation, an oleic acid solution was added into the prewarmed medium and was incubated under shaking for 6 h at 37°C. ARPE‐19 cells were treated with oleic acid (100, 250, 500, 1000 µM) for 24 h to induce a lipid‐overload model (Chang et al., 2020). The condition of 250 µM oleic acid for 24 h was selected for treating ARPE‐19 cells. ARPE‐19 cells were washed with PBS to remove oleic acid before adding EVs to the medium. Different concentrations of EVs (1 × 10^8^ EVs/mL, 1 × 10^9^ EVs/mL, 1×10^10^ EVs/mL) were used to treat the injured ARPE‐19 cells.

### 2.11 Cell viability measurement

The Cell Counting Kit‐8 (C0038; Beyotime Biotechnology) was used to measure cell viability. ARPE‐19 cells were washed thrice with PBS before incubation in DMEM supplemented with 10% CCK‐8 assay solution at 37°C for 45 min. Optical density was measured at 450 nm using a microplate reader (Varioskan Flash; Thermo Fisher Scientific).

### 2.12 Reactive oxygen species assay

Reactive oxygen species (ROS) in ARPE‐19 cells were detected using the 2,7‐dichlorodihydrofluorescein diacetate (DCFH‐DA) assay (S0033S, Beyotime Biotechnology). According to the manufacturer's protocol, ARPE‐19 cells were incubated with DCFH‐DA at a concentration of 10 µM at 37°C for 30 min. Finally, images were acquired at 488 nm using a confocal laser scanning microscope (LSM 800; Zeiss, Germany).

### 2.13 Lipid staining

Accumulated lipids were stained according to previously reported methods (Wang et al., 2022). Briefly, the ARPE‐19 cells were fixed with 4% PFA. Nile red (HY‐D0718; MedChemExpress) at 1 µM in PBS was added for staining at room temperature for 10 min. After washing thrice with PBS, the nuclei were counterstained with DAPI. The samples were viewed and photographed at 568 nm using a confocal laser scanning microscope (LSM 800; Zeiss).

### 2.14 Phagocytosis measurement

Following oleic acid or EV treatment, ARPE‐19 cells were incubated with the medium containing latex beads (L4655, FITC‐labelled, 1 µM; Sigma‐Aldrich). After 6 h of incubation, the ARPE‐19 cells were washed thrice with PBS to remove uningested latex beads. A confocal laser scanning microscope (LSM 800;  Zeiss) was used to acquire fluorescent images after staining the nuclei. Finally, the latex beads ingested by each ARPE‐19 cell were analysed with relative fluorescence intensity to quantify phagocytic capability.

### 2.15 PKH26 and MitoTracker staining

The RPC‐EVs were labelled with PKH26 following the manufacturer's instructions (MINI26; Sigma‐Aldrich). Briefly, EV pellets were resuspended in 1 mL diluent C before mixing with the stain solution (4 µL PKH26 Cell Linker in ethanol and 1 mL diluent C). After staining for 5 min at room temperature, 2 mL of 1% bovine serum albumin was added to the mixture, which was then diluted to 70 mL with PBS, followed by ultracentrifugation at 100,000 × *g* for 70 min. The EV pellets were then resuspended in PBS and were used for subsequent experiments. The same labelling procedure without EVs was performed as the negative control. According to the manufacturer's instructions, ARPE‐19 cells were incubated with MitoTracker Green (9074; Cell Signaling Technology, USA), which was added to the medium at a 100 nM concentration for 30 min to stain mitochondria. Images were captured at 561 nm (PKH26) and 488 nm (MitoTracker Green) using a confocal laser scanning microscope (AX confocal microscope; Nikon, Japan).

### 2.16 Statistical analysis

Data are presented as the mean ± SD for at least three biological samples. One‐way analysis of variance, followed by Tukey's multiple comparisons test, was conducted for multiple comparisons, and Benjamini–Hochberg‐corrected *t*‐test was used for diameter comparison between the hESC‐EV and hRPC‐EV groups using GraphPad Prism 9.2.0. Differences were considered statistically significant at *p* < 0.05.

## 3 RESULTS

### 3.1 Characteristics of hRPC‐EVs and hESC‐EVs

**FIGURE 1:**
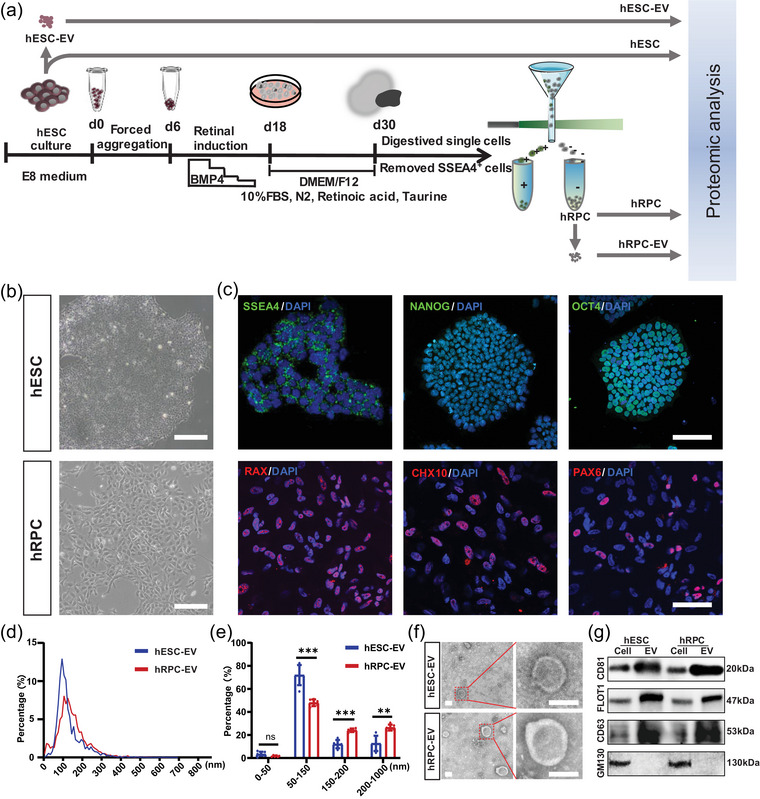
Isolation and characterisation of extracellular vesicles (EVs) derived from human embryonic stem cells (hESCs) and human retinal organoid‐derived retinal progenitor cells (hRPCs). (a) Induction of retinal organoids and schematic representation of the experimental design. (b) Typical morphology of hESCs and hRPCs under a light field. (c) Fluorescent staining of hESC markers (SSEA4, NANOG and OCT4) and hRPC markers (RAX, CHX10 and PAX6). (d, e) Size distribution and percentage of hESC‐EVs and hRPC‐EVs via nanoparticle tracking analysis. (f) Transmission electron micrographs showing the morphology of EVs derived from hESCs and hRPCs. (g) Identification of EV markers in hESCs, hRPCs, hESC‐EVs and hRPC‐EVs via western blotting. Data are presented as the mean ± SD, *n* = 6 (e). **p < 0.01; ***p < 0.001; ns, not significant (Benjamini‐Hochberg corrected *t*‐tests for e). Scale bar, 200 µm (b), 100 µm (c), 100 nm (f).

hRPC‐EVs and hESC‐EVs were isolated from hRPCs and hESCs, respectively (Figure 1a). To acquire hRPCs, hEROs were generated as described in our previous studies (Figure S1a–f) (Zeng et al., 2021; Zou et al., 2019). Retinal cell markers (CHX10, HuC/D, PAX6, and RAX) were positive in hEROs on day 30 (d30; Figure S1d–f). SSEA4^−^ hRPCs were isolated from d30 hEROs using FACS to remove undifferentiated ESCs and avoid tumorigenesis. The hESCs were collected as controls (Figure 1a). hRPCs were positive for retinal progenitor markers, such as RAX, PAX6 and CHX10, whereas hESCs expressed pluripotent markers, including SSEA4, NANOG and OCT4 (Figure 1b,c). Both EVs were isolated from hRPCs and hESCs via differential centrifugation; most particle sizes were in the range of 50–250 nm and peaked at approximately 100 nm in diameter when detected using nanoparticle tracking analysis (NTA; Figure 1d,e, Figure S1g,h). The diameter of more than 75% of hESC‐EVs ranged from 50 to 150 nm, whereas the percentage of hRPC‐EVs with a diameter of 50–150 nm was approximately 50%. The percentage of hRPC‐EVs larger than 150 and 200 nm in diameter was greater than that of hESC‐EVs (Figure 1e). TEM confirmed that both types of EVs had a typical spheroid morphology (Figure 1f). Compared with those of cells, the specific EV markers, CD81, FLOT1 and CD63, were highly expressed in hRPC‐EVs and hESC‐EVs, according to western blotting analysis (Figure 1g, Figure S9a–c). The negative marker, GM130, was absent in the EVs. Using NTA, TEM and western blotting, EVs were successfully isolated from hRPCs and hESCs. Together, both EVs were highly heterogeneous, and the proportion of medium/large EVs (>200 nm) was higher in hRPC‐EVs.

### 3.2 Extracellular vesicles derived from hESCs and hRPCs exhibit remarkable dissimilarity

**FIGURE 2:**
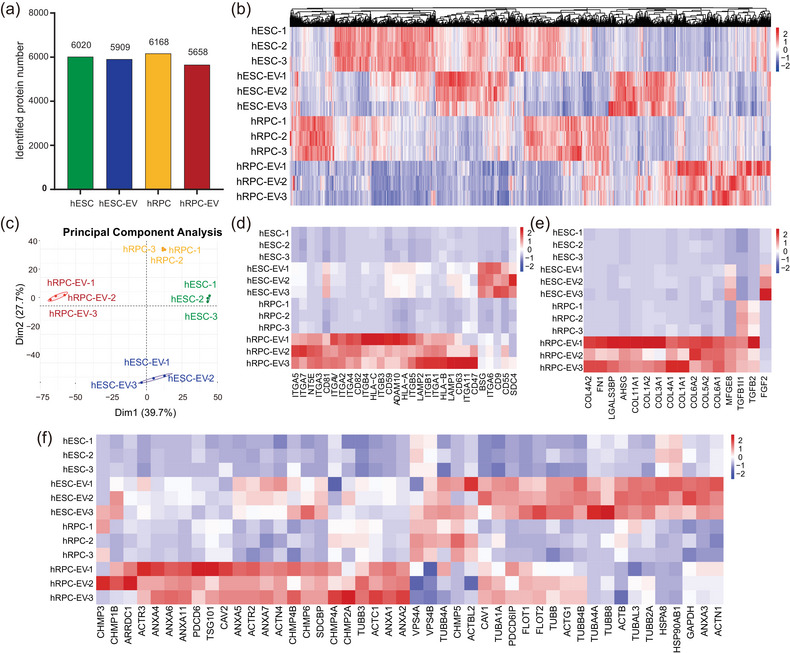
Proteomic analysis of hESCs‐EVs, hRPC‐EVs, and their cells of origin. (a) Numbers of identified proteins in the four groups. (b) Expression heatmap of all identified proteins in the four groups. (c) Protein quantitative principal component analysis (PCA) of all samples. (d–f) Expression heatmap of three categories of EV‐associated markers in all groups. Transmembrane or GPI‐anchored proteins (d). Secreted or luminal proteins recovered in EVs (e). Cytosolic or periplasmic proteins (f).

In‐depth proteomics was performed to determine the specific protein contents of both EV types (Tai et al., 2022). More than 90% of peptides comprised 7–30 amino acids (Figure S2a). Andromeda analysis showed that the median score was 85.12 with high quality (Figure S2b), meeting the quality control of LC‐MS/MS. The number of identified proteins in hESCs, hESC‐EVs, hRPCs and hRPC‐EVs was 6020, 5909, 6168 and 5658, respectively (Figure 2a). The protein expression heatmap of all four groups and the principal component analysis plot indicated that the three biological replicates of each group were homogeneous, confirming the repeatability and reliability of the data (Figure 2b,c). However, the proteomic expression in each group distinctly differed (Figure 2b). According to the minimal information for studies of EVs 2018 (Théry et al., 2018), three categories of EV marker proteins (transmembrane or GPI‐anchored proteins, secreted or luminal proteins recovered in EVs, and cytosolic or periplasmic proteins) were analysed (Figure 2d–f). Notably, most transmembrane or GPI‐anchored and cytosolic or periplasmic proteins were more significantly enriched in hESC‐EVs and hRPC‐EVs than in hESCs and hRPCs, respectively (Figure 2d,f). However, most secreted or luminal proteins were specifically enriched in hRPC‐EVs (Figure 2e). Interestingly, both EVs upregulated EV markers, such as CD81, CD63, FLOT1 and FLOT2. However, hESC‐EVs and hRPC‐EVs were differently enriched with specific EV markers. For instance, CD9, ACTB, GAPDH, HSPA8 and TUBA1C were highly expressed in hESC‐EVs while weakly expressed in hRPC‐EVs, whereas TSG101, FN1 and members of the collagen family showed the opposite pattern (Figure 2d–f). This indicates that the protein profiles of hESC‐EVs and hRPC‐EVs exhibited remarkable dissimilarities.

### 3.3 Comparison of proteomic results between hESC and hESC‐EV groups

**FIGURE 3:**
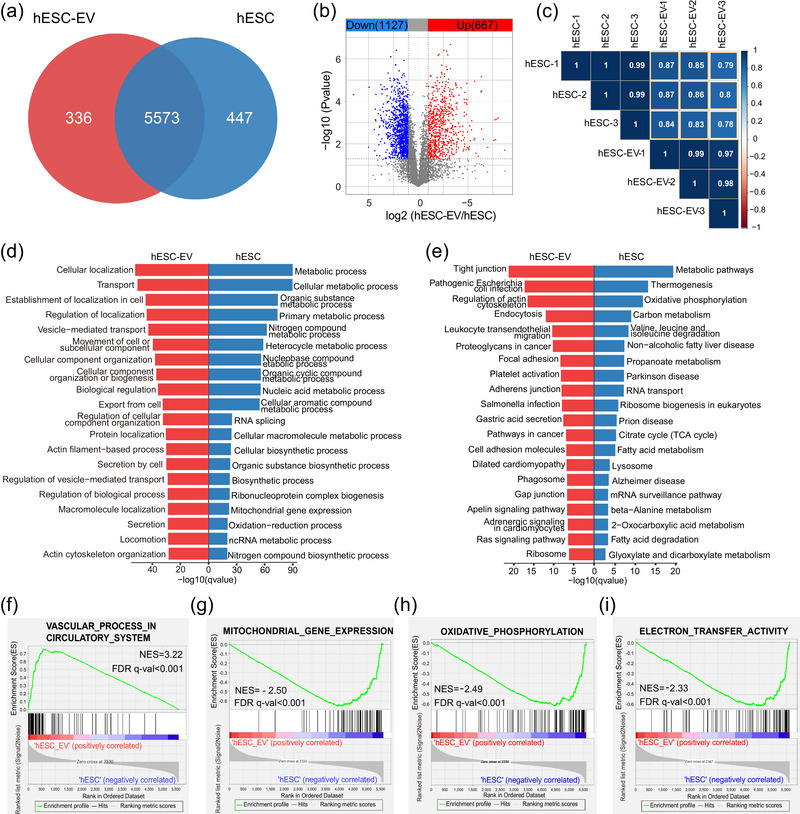
Comparison of proteomic results between hESC and hESC‐EV groups. (a) Venn diagram showing identified proteins in the hESC and hESC‐EV groups. (b) Volcano plot of highly and lowly expressed proteins in the hESC‐EV group compared with those in the hESC group. (c) Pearson correlation heatmap of the hESC and hESCs‐EV proteome. (d) Gene ontology (GO; Biological Process) analysis of highly expressed proteins in hESC‐EVs (red columns) and hESCs (blue columns). (e) KEGG analysis of highly expressed proteins in hESC‐EVs and hESCs. (f) Significantly upregulated gene set in hESC‐EVs compared with that in hESCs determined by Gene Set Enrichment Analysis (GSEA). (g–i) Significantly downregulated gene sets in hESC‐EVs compared with those in hESCs determined by GSEA.

First, the protein contents of hESCs and hESC‐EVs were compared. A total of 5573 proteins were identified in both hESC‐EVs and hESCs, whereas 447 and 336 proteins were unique to hESCs and hESC‐EVs, respectively (Figure 3a). There were 1794 proteins recognised as DEPs between hESC‐EVs and hESCs, of which 667 proteins were highly expressed in hESC‐EVs and 1127 proteins were highly expressed in hESCs (Figure 3b). Correlation analysis showed that the Pearson correlation coefficient between hESCs and hESC‐EVs ranged from 0.78 to 0.87, suggesting that the protein content in hESC‐EVs was highly analogous with that in hESCs (Figure 3c).

We firstly focused on highly expressed DEPs in hESC‐EVs, and GO analysis showed that these proteins were closely related to cell secretion and vesicle‐mediated transport, reflecting the core properties of EVs (Figure 3d, Table S1). In addition, KEGG pathway analysis confirmed that the pathways involved in EV biogenesis, secretion and transport, such as endocytosis, phagosome and focal adhesion, significantly differed between hESC‐EVs and hESCs (Figure 3e, Table S1). Consistent with a previous report (Granger et al., 2014), proteins related to the regulation of the actin cytoskeleton were also highly enriched in hESC‐EVs, as the budding and release of EVs are associated with actin polymerisation (Figure 3e). The top‐listed GO and KEGG pathways demonstrated that DEPs with low expression in hESC‐EVs were closely related to metabolic processes, demonstrating that metabolism‐related proteins were not selectively enriched in hESC‐EVs (Figure 3d,e, Table S2). Intriguingly, proteins associated with energy metabolism (oxidative phosphorylation and the citrate cycle) and fatty acid metabolism were not abundant in hESC‐EVs (Figure 3e). Additionally, the PPI network showed that hESC‐EV‐enriched proteins were related to vesicle transport, system development, vasculature development, and SLC‐mediated transport (Figure S2). To further support this conclusion, GSEA was performed to determine whether a defined set of genes encoding proteins significantly differed between hESCs and hESC‐EVs. This revealed that the vascular process in the circulatory system set was upregulated in hESC‐EVs compared to that in hESCs (Figure 3f, Table S3). Proteins related to angiogenesis, such as CEACAM1, PTPRJ and SLC family members, were enriched in hESC‐EVs (Figure 3f, Table S3). In addition, gene sets involved in energy metabolism, such as mitochondrial gene expression, electron transfer activity and oxidative phosphorylation, were downregulated in hESC‐EVs compared to those in hESCs (Figure 3g–i). Taken together, protein cargoes related to angiogenesis was selectively loaded into hESC‐EVs, whereas those related to metabolism, such as mitochondria and energy metabolism, were possibly excluded from hESC‐EVs during EV generation.

### 3.4 Comparison of proteomic results between hRPC and hRPC‐EV groups

**FIGURE 4:**
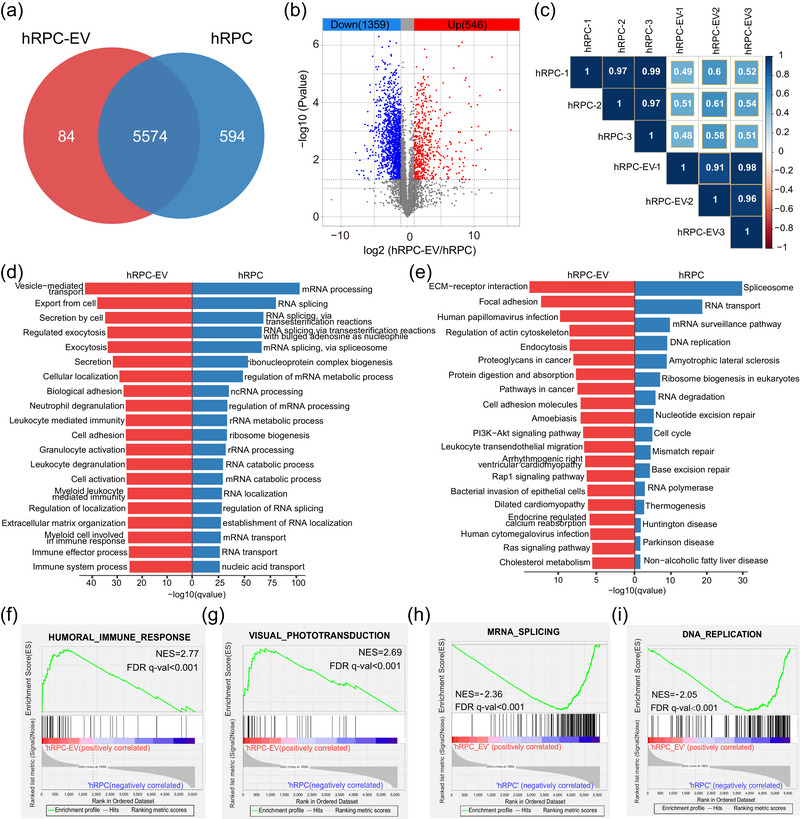
Comparison of proteomic results between hRPC and hRPC‐EV groups. (a) Venn diagram showing identified proteins in the hRPC and hRPC‐EV groups. (b) Volcano plot of highly and lowly expressed proteins in the hRPC‐EV group compared with those in the hRPC group. (c) Correlation heatmap of the hRPC and hRPC‐EV proteome. (d) GO (Biological Process) analysis of highly expressed proteins in hRPC‐EVs (red columns) and hRPCs (blue columns). (e) KEGG analysis of highly expressed proteins in hRPC‐EVs and hRPCs. (f and g) Significantly upregulated gene sets in hRPC‐EVs determined by GSEA. (h and i) Significantly downregulated gene sets in hRPC‐EVs determined by GSEA.

We then compared the protein content of hRPC‐EVs and hRPCs. There were 5574 proteins present in both hRPC‐EVs and hRPCs, whereas 594 and 84 proteins were unique to hRPCs and hRPC‐EVs, respectively (Figure 4a). The total number of identified DEPs was 1905. Among them, 546 and 1359 proteins were markedly enriched in hRPC‐EVs and hRPCs, respectively (Figure 4b). Notably, compared with that of hESC‐EVs and hESCs, a lower correlation (correlation coefficient ranged from 0.48 to 0.61) between hRPC‐EVs and hRPCs was observed, suggesting a more selective process during hRPC‐EV secretion (Figure 4c).

GO and KEGG analysis were performed to identify the pathways involved in high‐ and low‐expression proteins in hRPC‐EVs relative to hRPCs (Figure 4d,e). For GO enrichment analysis of the enriched proteins, in addition to EV transport and secretion processes, immune‐related items (immune system processes, immune effector progress and leukocyte‐mediated immunity) were enriched (Figure 4d, Table S4). KEGG analysis revealed that the PI3K‐AKT signalling pathway, a classical immune regulator, was enriched in hRPC‐EVs (Figure 4e, Table S4). Meanwhile, GSEA highlighted the human immune response as the upregulated pathway in hRPC‐EVs (Figure 4f, Table S5). Among enriched proteins, ITGA4, CD55 and SERPING1 were involved in regulating immune cell behavior (Figure 4f, Table S5) (Bowman et al., 2016; Gelderman et al., 2004; Ni et al., 2022). Interaction analysis demonstrated that proteins highly expressed in hRPC‐EVs were related to EV transport, immune system processes, axon guidance and retinoid metabolism (Figure S3). Based on the GSEA, visual phototransduction‐related proteins were loaded and upregulated in hRPC‐EVs (Figure 4g). Furthermore, GO analysis demonstrated that the proteins absent from hRPC‐EVs were mainly related to cell nuclei rather than being proteins related to mitochondria and energy metabolism, unlike in hESC‐EVs (Figure 4d, Table S6). KEGG analysis also indicated that cell cycle‐related proteins were not enriched in hRPC‐EVs (Figure 4e, Table S6). Meanwhile, GSEA indicated that DNA replication‐and gene transcription‐related items (DNA replication, mRNA splicing, etc.) were expressed at lower levels in hRPC‐EVs than in hRPCs (Figure 4h,i). Collectively, these results reflected the retinal developmental, immunomodulatory and low proliferative potential of hRPC‐EVs.

### 3.5 Proteins in hESC‐EVs exhibit a stronger correlation with cell proliferation than those in hRPC‐EVs

**FIGURE 5:**
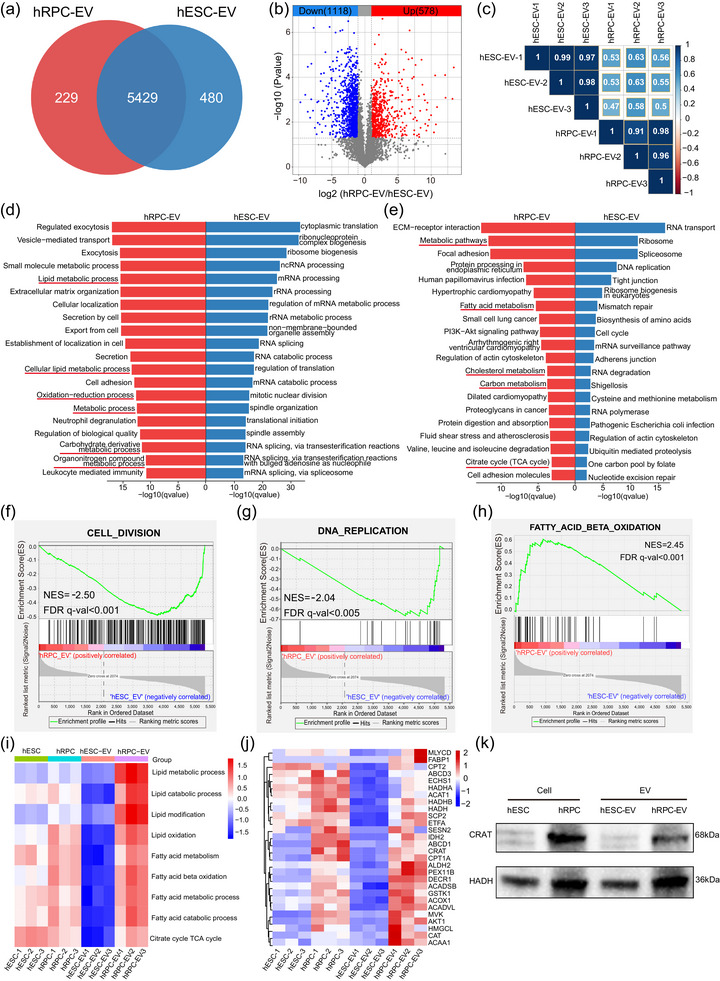
Comparison of proteomic results between the hRPC‐EV and hESC‐EV groups. (a) Venn diagram showing identified proteins in the hESC‐EV and hRPC‐EV groups. (b) Volcano plot of highly and lowly expressed proteins in the hESC‐EV group compared with those in the hRPC‐EV group. (c) Correlation heatmap of the hESC‐EV and hRPC‐EV proteome. (d) GO (Biological Process) analysis of highly expressed proteins in hRPC‐EVs (red columns) and hESC‐EVs (blue columns). (e) KEGG analysis of highly expressed proteins in hRPC‐EVs and hRPC‐EVs. (f and g) Significantly upregulated gene sets in hESC‐EVs determined by GSEA. (h) GSEA of fatty acid β‐oxidation pathways in hRPC‐EVs and hESC‐EVs. (i) Score heatmap of lipid and fatty acid metabolism‐related pathways in all four groups determined by single sample GSEA. (j) Expression heatmap of proteins related to fatty acid β‐oxidation in all groups. (k) Expression of CRAT and HADH proteins through western blotting.

For EV protein features, hESC‐EVs and hRPC‐EVs were compared. There were 5429 proteins expressed in both EVs, of which 480 were only expressed in hESC‐EVs and 229 only detected in hRPC‐EVs (Figure 5a). Although hRPC‐EVs shared 95.9% of their proteins with hESC‐EVs, the protein levels in hESC‐EVs and hRPC‐EVs remarkably differed. Differential expression analysis showed that 1118 and 578 proteins were recognised as highly expressed DEPs in hESC‐EVs and hRPC‐EVs, respectively (Figure 5b). The Pearson correlation coefficient ranged from 0.47 to 0.63, suggesting a significant difference in protein levels and types in hESC‐EVs and hRPC‐EVs (Figure 5c).

Based on GO and KEGG enrichment analyses of the highly expressed DEPs in hESC‐EVs, several pathways associated with RNA processing were identified (Figure 5d,e, Table S7). Moreover, pathways related to cell division and proliferation, such as mitotic nuclear division, spindle organisation, cell cycle and DNA replication, were enriched in hESC‐EVs than in hRPC‐EVs (Figure 5d,e, Table S7). GSEA further confirmed that hESC‐EVs maintained higher protein levels in the cell division and DNA replication gene sets (Figure 5f,g). Similarly, the pathway interaction network showed that chromosome‐, nuclear‐ and cell proliferation‐related pathways were enriched in hESC‐EVs, including chromium organisation, mitotic spindle organisation and cell division (Figure S4a). In addition, mRNA metabolic process and ribosome biogenesis were enriched in hESC‐EVs (Figure S4b,c). Overall, hESC‐EV proteins strongly correlated with cell division and proliferation, which likely contributed to stemness maintenance. However, this also raises safety concerns regarding the application of hESC‐EVs in patients with RD.

### 3.6 hRPC‐EVs potentially regulate lipid metabolism

To identify the features of hRPC‐EVs, GO and KEGG enrichment analyses for highly expressed DEPs in hRPC‐EVs indicated that the metabolic process was the top‐listed pathway (Figure 5d,e, Table S8). Moreover, several metabolic pathways were enriched in the GO analysis, including the organonitrogen compound metabolic process, lipid metabolic process and carbohydrate derivative metabolic process (Figure 5d, Table S8). Carbon metabolism, fatty acid metabolism and cholesterol metabolism, as well as citrate cycle pathways, which are closely associated with glucose and lipid metabolism, confirmed by the KEGG pathway analysis (Figure 5e, Table S8). PPI network analysis showed that the highly expressed proteins in hRPC‐EVs were related to metabolic processes, system development, leukocyte‐mediated immunity and PI3K‐AKT signalling (Figure S5), further confirming the importance of metabolic processes in hRPC‐EVs. Pathway interaction network analysis showed that pathway clusters in highly expressed DEPs of hRPC‐EVs mainly comprised four components: (1) cell adhesion and nervous system development (e.g., integrin‐mediated signalling pathway, neurogenesis and cell morphogenesis; Figure S6a); (2) Metabolic processes (e.g., lipid metabolic process, carbohydrate derivative metabolic process and organic hydroxyl compound metabolic process; Figure S6b,c); (3) Immune regulation and cell secretion (e.g., leukocyte‐mediated immunity and regulated exocytosis; Figure S6d); (4) Wound healing (Figure S6e). As described above, immune‐related pathways were upregulated in hRPC‐EVs (Figure 5d,e, Figures S5 and S6d), confirming their potential for immune regulation, as suggested by our previous studies (Bian et al., 2020).

Owing to the metabolic processes, especially lipid metabolism, with the critical functional protein pathway in hRPC‐EVs, we further analysed lipid metabolism. GSEA showed the upregulation of fatty acid β‐oxidation with a normalised enrichment score of 2.45 and FDR <0.001 (Figure 5h). Additionally, results of a single sample GSEA indicated that the scores of lipid and fatty acid metabolism‐related pathways were the highest in the hRPC‐EV group relative to those of the other three groups (Figure 5i). We focused on specific proteins, including several transporters and vital enzymes involved in fatty acid metabolism (Figure 5j). Compared with those in hESCs, the levels of fatty acid metabolism‐related proteins in hRPCs were higher, suggesting that the activity of fatty acid metabolism gradually increased during retinal differentiation (Figure 5j). Similarly, fatty acid metabolism‐related proteins were not enriched in hESC‐EVs during generation. In contrast, hRPC‐EVs loaded proteins to metabolise fatty acids during production, including enzymes such as HADH, CRAT, CPT1A and ACADVL, and transporters such as FABP1 and ABCD1. Additionally, fatty acid metabolism‐related proteins were expressed at higher levels in the hRPC‐EVs than in hESC‐EVs (Figure 5j). MS results were confirmed through western blotting, which showed that the proteins related to fatty acid oxidation (CRAT and HADH) were highly expressed in hRPCs and hRPC‐EVs (Figure 5k, Figure S9d,e). Collectively, these results demonstrate that hRPC‐EVs are enriched with fatty acid‐specific transporters and enzymes related to fatty acid metabolism.

### 3.7 Lipid‐overloaded cytotoxicity of retinal pigment epithelium cells is diminished by hRPC‐EVs through the regulation of fatty acid metabolism

**FIGURE 6:**
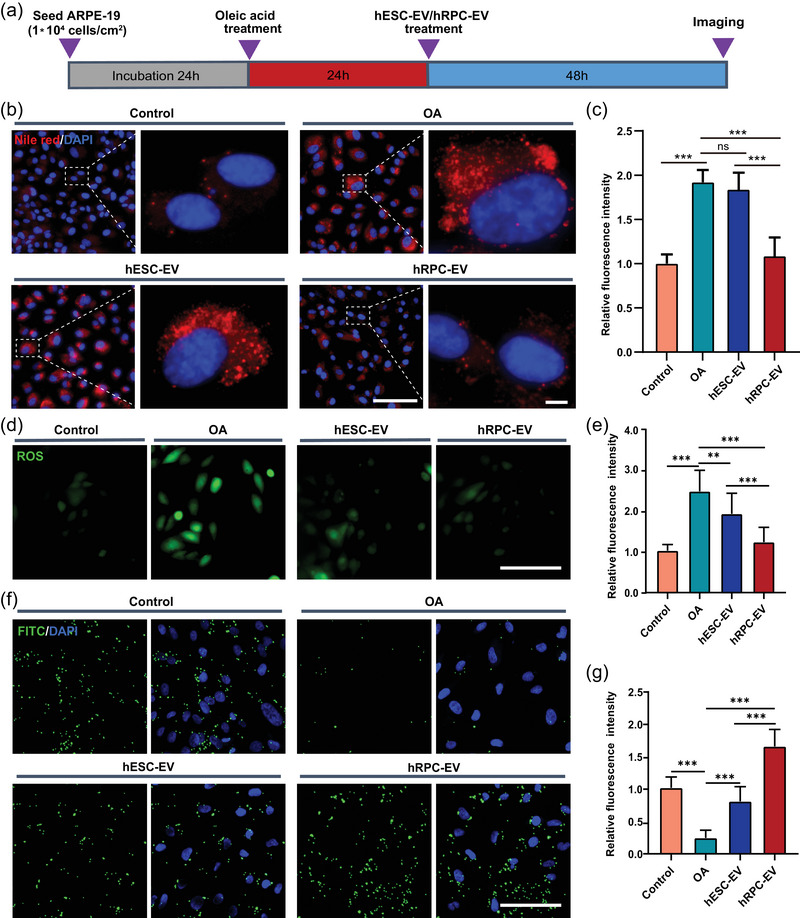
Effect of hESC‐EVs and hRPC‐EVs on oleic acid treated ARPE‐19 cells. (a) Experimental design. (b, c) Representative images of Nile red staining and relative fluorescence intensity analysis of ARPE‐19 cells in the Control, OA, hESC‐EV, and hRPC‐EV groups. (d) Representative images of reactive oxygen species (ROS) staining in all four groups. (e) Relative fluorescence intensity analysis of ROS. (f) Representative images showing the uptake of FITC^+^ beads by ARPE‐19 cells. (g) Relative fluorescence intensity analysis of FITC‐labelled beads. Data are presented as the mean ± SD, *n* = 6 (c, e, g). **p < 0.01; ***p < 0.001; ns, not significant (one‐way ANOVA for c, e, g). Scale bars: 100 µm (b, d, f), 5 µm (enlarged image of b). OA: oleic acid.

To investigate the potential of hRPC‐EVs in preventing RPE degeneration and treating AMD, a lipid‐overload model of RPE cells was established by adding oleic acid (Agrón et al., 2021; Lita et al., 2021; Yako et al., 2022). Treatment with 250 µM oleic acid for 24 h led to a 25% decline in cell viability, whereas higher concentrations (500 and 1000 µM) resulted in more than 80% cell death (Figure S7a,b). Nile red staining revealed intracellular lipid accumulation in oleic acid‐treated ARPE‐19 cells (Figure S7c,d). To mimic the lipotoxicity of ARPE‐19 cells without causing severe cell death, 250 µM oleic acid was selected for subsequent experiments. After oleic acid treatment, hRPC‐EVs or hESC‐EVs at different concentrations (1 × 10^8^, 1 × 10^9^ and 1 × 10^10^ EVs/mL) were co‐cultured with ARPE‐19 cells for another 48 h (Figure 6a, Figure S7e). Cell viability was enhanced at a concentration of 1×10^9^ EVs/mL in both EV groups and was even greater at a concentration of 1 × 10^10^ EVs/mL (Figure S7e). The hRPC‐EV treatment (1 × 10^10^ EVs/mL) significantly delayed lipid deposition, as evidenced by Nile red staining, and hESC‐EV treatment had a weak impact on lipid deposition (Figure 6b,c), suggesting that hRPC‐EVs played a unique role in regulating lipid metabolism. Furthermore, ROS production increased significantly in the oleic acid‐treated group and was significantly reversed by both hESC‐EV and hRPC‐EV treatments (Figure 6d,e). Notably, hRPC‐EVs exhibited better ability to reverse ROS, potentially indicating the inhibition of oxidative stress. FITC bead‐based phagocytosis measurements showed that the oleic acid treatment impaired the phagocytosis of ARPE‐19 cells. hRPC‐EVs reversed ARPE‐19 cell dysfunction in engulfing FITC‐labelled beads more effectively than hESC‐EVs (Figure 6f,g).

**FIGURE 7:**
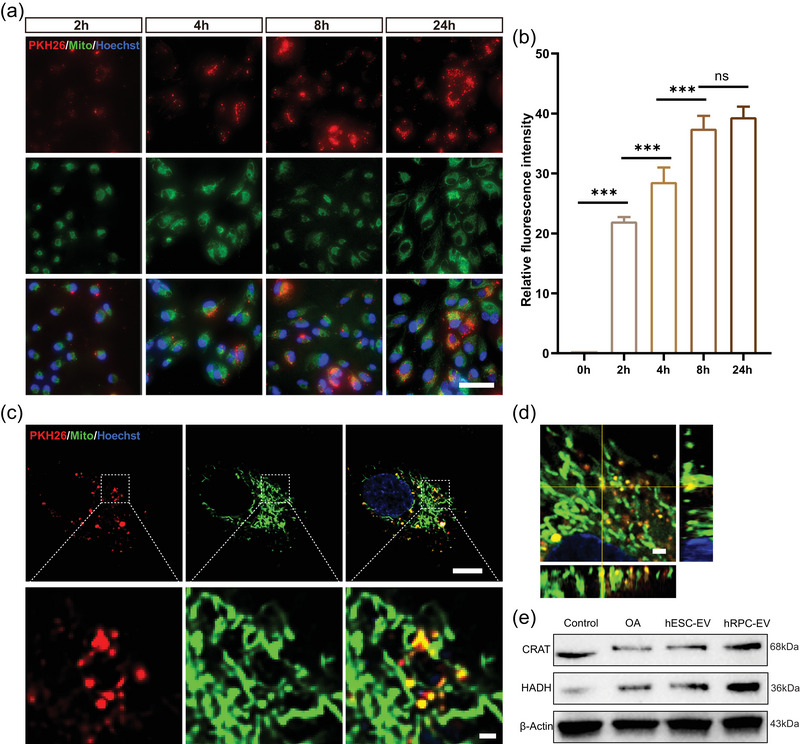
RPC‐EVs were internalized and integrated into the mitochondrial network of oleic acid‐treated ARPE‐19 cells. (a) Internalisation of PKH26‐labelled RPC‐EVs in OA‐treated ARPE‐19 cells at different timepoints. (b) Relative fluorescence intensity analysis of PKH26‐labelled RPC‐EVs. (c) Internalised RPC‐EVs integrated into the mitochondrial network of ARPE‐19 cells at 24 h. (d) Orthogonal image of RPC‐EV integration into the mitochondrial network of ARPE‐19 cells. (e) Western blotting results of lipid metabolism‐related proteins in oleic acid treated ARPE‐19 cells. Data are presented as the mean ± SD, *n* = 6 (b). **p < 0.01; ***p < 0.001; ns, not significant (one‐way ANOVA for b). Scale bars: 50 µm (a), 10 µm (c), 1 µm (enlarged image of c, d).

Abnormal mitochondrial fatty acid β‐oxidation can lead to lipid accumulation (Tan et al., 2020). MitoTracker Green was used to label the mitochondria of ARPE‐19 cells, and PKH26 was used to label hRPC‐EVs. Over time, hRPC‐EV uptake by oleic acid‐treated ARPE‐19 cells increased, reaching relative saturation after 8 h (Figure 7a,b). The negative control experiment (the comparable labelling procedure without EVs) was carried out and almost no positive fluorescent signals were observed in ARPE‐19 cells (Figure S8). Furthermore, the ingested EVs were infused into the mitochondrial network of ARPE‐19 cells at 24 h (Figure 7c,d, Movie S1). As hRPC‐EVs are enriched in lipid regulatory‐associated proteins (Figure 5d,e,h–k, Figures S5 and S6), we investigated their effect on fatty acid β‐oxidation. Notably, the expression of fatty acid β‐oxidation‐related proteins (CRAT and HADH) in oleic acid‐treated ARPE‐19 cells increased after hRPC‐EV treatment, as determined by western blotting (Figure 7e, Figure S9f,g), suggesting that hRPC‐EVs may improve the ability of RPE cells to degrade fatty acids by enhancing mitochondrial fatty acid β‐oxidation.

## 4 DISCUSSION

EVs, considered vital mediators of cell‐cell communication and a promising source for RD treatment, vary in composition depending on the cell source (van Niel et al., 2022). Therefore, clarifying the components of EVs is crucial for understanding their potential in cell‐free therapy for RD. To the best of our knowledge, our study is one of the first studies to uncover the protein composition of EVs derived from organoid‐derived hRPCs using in‐depth proteomic analysis. Our findings indicated that hRPC‐EVs have a greater difference in protein composition than hESC‐EVs or their cells of origin. This demonstrated that hESC‐EVs were more likely enriched with angiogenic than metabolic proteins, whereas hRPC‐EV‐enriched proteins were closely associated with immune regulation and retinal development and rarely associated with the cell cycle. Additionally, hESC‐EV proteins exhibited a stronger correlation with cell proliferation than hRPC‐EVs, indicating a potential risk of tumorigenesis. More importantly, proteomic analysis revealed that hRPC‐EVs maintained a unique capacity to regulate lipid metabolism compared with hESC‐EVs. It was further confirmed that hRPC‐EVs could reduce lipid deposits, inhibit lipotoxicity, and improve ARPE‐19 cell phagocytosis in vitro, probably through integrating into the mitochondrial network of ARPE‐19 cells and increasing the expression of mitochondrial fatty acid β‐oxidation‐related proteins. Overall, this study provides new insights into the application of organoid‐derived hRPC‐EVs for treating RD characterised by lipid deposition in the RPE.

According to MISEV2018, EVs can be classified into small (<200 nm) and medium/large EVs (>200 nm) depending on their size (Théry et al., 2018). In this study, we found that hESCs and hRPCs secreted both small and medium/large EVs. The proportion of EVs >200 nm was greater in hRPC‐EVs (Figure 1d,e), which is in line with the suggestion that cell‐secreted EVs have different sizes depending on the differentiation stage of their cells of origin (Louro et al., 2022). Of note, hESC‐EVs and hRPC‐EVs were enriched with specific EV markers. Owing to the critical role of proteins such as CD81, TSG101, CD9 and CD63 in regulating EV generation (van Niel et al., 2022), the biogenesis and secretion of hESC‐EVs and hRPC‐EVs likely rely on different mechanisms. During EV biogenesis, the cargo is selectively loaded into the EVs or merely reflects the characteristics of the cytoplasmic content. Remarkably, it was previously reported that more than 100 proteins were abundant in adipose‐derived MSC‐isolated EVs compared to those in the cell itself, suggesting that the loading of EVs is not simply a diffusion process but involves active and specific trafficking (Eirin et al., 2016). To reveal the protein signature of hRPC‐EVs, we performed an in‐depth proteomic analysis and found that hRPC‐EVs and their respective cells of origin showed a lower correlation than did hESC‐EVs and their respective cells of origin. Furthermore, hRPC‐EVs contained an abundance of secreted or luminal proteins. hESC‐EVs seemed to exhibit higher protein conservation, and hRPC‐EVs might have the potential to target specific functional proteins. Similarly, it was previously reported that the correlation between iPSCs and their EVs was higher than that between iPSC‐derived MSCs and their EVs (La Greca et al., 2018), suggesting that the selection of the EV cargo is enhanced during the differentiation of iPSCs into MSCs. Our results demonstrated that hESC‐EVs and hRPC‐EVs presented high dissimilarity, similar to reports showing that the content of EVs was distinct during iPSC‐cardiomyocyte and MSC differentiation (Louro et al., 2022; Wang et al., 2018). Thus, we conjecture that as pluripotent stem cells differentiate into tissue‐specific or unipotent stem cells, their capacity to select cargo for EVs might be enhanced.

The hESC‐EVs have been used to treat various systemic diseases but are rarely used in the treatment of retinal diseases because of their potential tumorigenic risk. Here, we performed proteomic analysis of hESC‐EVs and their cells of origin. Compared to that in hESCs, hESC‐EVs were enriched in proteins related to angiogenesis. Several research groups have reported that ESC‐EVs or iPSCs facilitate angiogenesis by enhancing vascular endothelial cell proliferation and tube formation speed in kidney injury, pressure ulcers and heart failure (Chen et al., 2019; Pang et al., 2021; Yu et al., 2021). Therefore, according to our results, angiogenesis‐promoting proteins are likely to be involved in vascular repair. Proteins involved in cell proliferation were enriched in hESC‐EVs. Thus, hESCs likely maintain their stem cell phenotype through EVs (Hur et al., 2021). In addition, compared with those of hRPC‐EVs, hESC‐EV proteins more strongly correlated with cell division and proliferation (Figure 5). Likewise, hESC‐EVs were found to promote the reprogramming of retinal Müller glial cells, which might be partly mediated by pluripotent protein cargo (Peng et al., 2018; Zhou et al., 2016). In this study, the viability of oleic acid‐treated RPE cells also increased in the hESC‐EV group without lipid clearance, likely due to the proliferation‐promoting ability of hESC‐EVs. However, unlimited proliferation and facilitation of vascular formation are typical tumorigenic features consistent with the characteristics of hESC‐EVs. Although there is no evidence that hESC‐EVs cause tumor formation, this should be evaluated more carefully in clinical trials. In contrast to that of hESC‐EVs, proteomic analysis of hRPC‐EVs revealed that the enriched proteins were not associated with cell proliferation, indicating that hRPC‐EVs may be safer for clinical treatment.

Proteins related to visual system development were abundant in hRPC‐EVs, reflecting the role of RPCs in retinal formation. The interactions between retinal cells and secreted mediators are vital for retinal development. RPC‐released EVs are critical factors driving RPC proliferation and differentiation (Zhou et al., 2018, 2021). Nonetheless, whether these proteins play a pivotal role in neuroregeneration during retinal damage warrants further investigation. In our previous study, EVs isolated from hRPCs were shown to regulate the inflammatory response by suppressing microglial activation in RD models (Bian et al., 2020; Zou et al., 2019); however, the underlying mechanism was unclear. Recently, it has been demonstrated that material transfer from stem cell‐derived donor cells to host cells may play a key role in stem cell‐based medicine (Santos‐Ferreira et al., 2016; Singh et al., 2016). In the non‐end‐stage degenerative or intact retina, most donor cells were observed to transfer RNA or proteins into host cells without nuclear fusion, possibly mediated through EVs (Kalargyrou et al., 2021; Pearson et al., 2016). In the current study, we discovered that hRPC‐EVs were enriched in proteins that regulate immune cell behavior (e.g., ITGA4, CD55 and SERPING1), which may play a role in regulating microglia to delay photoreceptor apoptosis, thus enhancing our understanding of the therapeutic mechanism of stem cell therapy.

RD in AMD and RP is caused by diverse genetic defects and environmental factors, with abnormal lipid metabolism being a common pathological feature (Khan et al., 2016). AMD, which causes irreversible blindness and affects 30 million older adults, is characterised by lipid accumulation and RPE cell dysfunction (Ren et al., 2022; Tan et al., 2020). RPE cells play a crucial role in the regulation of retinal lipid metabolism and their dysfunction leads to retinal photoreceptor damage and degeneration. Among various lipid components, fatty acids, especially oleic acid, are present in the subretinal space and recognised as critical risk factors for AMD (Agrón et al., 2021). Consistently, an overload of oleic acid causes lipid deposition, dysfunction and a decline in RPE cell viability (Yako et al., 2022). Thus, recovering the function of lipid metabolism in RPE cells and clearing lipids deposited in the subretinal space are potential treatments for AMD (Landowski & Bowes Rickman, 2021; Ren et al., 2022). Here, we showed that oleic acid overload caused lipid deposition, as well as impaired viability and phagocytic function, in RPE cells. Strikingly, hRPC‐EV treatment alleviated lipid deposition and lipotoxicity in RPE cells, consistent with the prediction of proteomic analysis. RPE cells maintain lipid metabolism homeostasis in the retina through the use of lipid metabolism‐related enzymes, with mitochondrial fatty acid β‐oxidation being a major pathway for fatty acid degradation (Landowski & Bowes Rickman, 2021). In this study, we further demonstrated that hRPC‐EVs integrated into the mitochondrial network of RPE cells and increased the expression of fatty acid β‐oxidation‐related proteins (CRAT and HADH) in oleic acid‐treated ARPE‐19 cells after treatment with hRPC‐EVs, likely contributing to the clearance of excess lipids. However, the effects of hRPC‐EVs on lipid deposition in RPE cells lack confirmation in animal models or clinical trials. It is not clear whether hRPC‐EVs will markedly reduce the formation of larger drusen, which is composed of deposited lipids, and is a typical pathological change in AMD. Further investigation are required to elucidate the mechanisms of hRPC‐EVs on lipid deposition in RPE cells.

Overall, we identified the compositions of hESC‐EV and hRPC‐EV protein cargo with in‐depth label‐free proteome analysis. Our results demonstrate that hESC‐EVs are appropriate for angiogenesis in vascular injury‐related diseases and also raise concerns about the risk of abnormal proliferation and tumor occurrence in their application. However, hRPC‐EVs do not contain similar risk proteins and maintain unique proteins for improving lipid metabolism and protecting RPE cells from lipid cytotoxicity, which is promising to treat RD by exerting its lipid regulatory mechanism.

## AUTHOR CONTRIBUTIONS

Haiwei Xu, Ting Zou and Xiaotang Fan conceived, designed and supervised the project, revised the manuscript; Hui Gao, Yuxiao Zeng and Xiaona Huang performed experiments, analyzed data; Hui Gao and Ting Zou wrote the manuscript; Luodan A, Xi Lin, and Jing Gong assisted with experiments; Qingle Liang and Jing Xie revised the manuscript.

## CONFLICT OF INTEREST STATEMENT

The authors declare that they have no conflicts of interest.

## Supporting information

Supplementary Information

Supplementary Information

Supplementary Information

Supplementary Information

Supplementary Information

Supplementary Information

Supplementary Information

Supplementary Information

Supplementary Information

Supplementary Information
