# Supplementary material for: Extracellular vesicles from organoid‐derived human retinal progenitor cells prevent lipid overload‐induced retinal pigment epithelium injury by regulating fatty acid metabolism
Source: J Extracell Vesicles. 2023 Dec 27;13(1):e12401. doi: 10.1002/jev2.12401 (PMC10752800; doi:10.1002/jev2.12401)
Supplement: Supplementary file 2 — Supplementary Information [file JEV2-13-e12401-s009.docx]

c
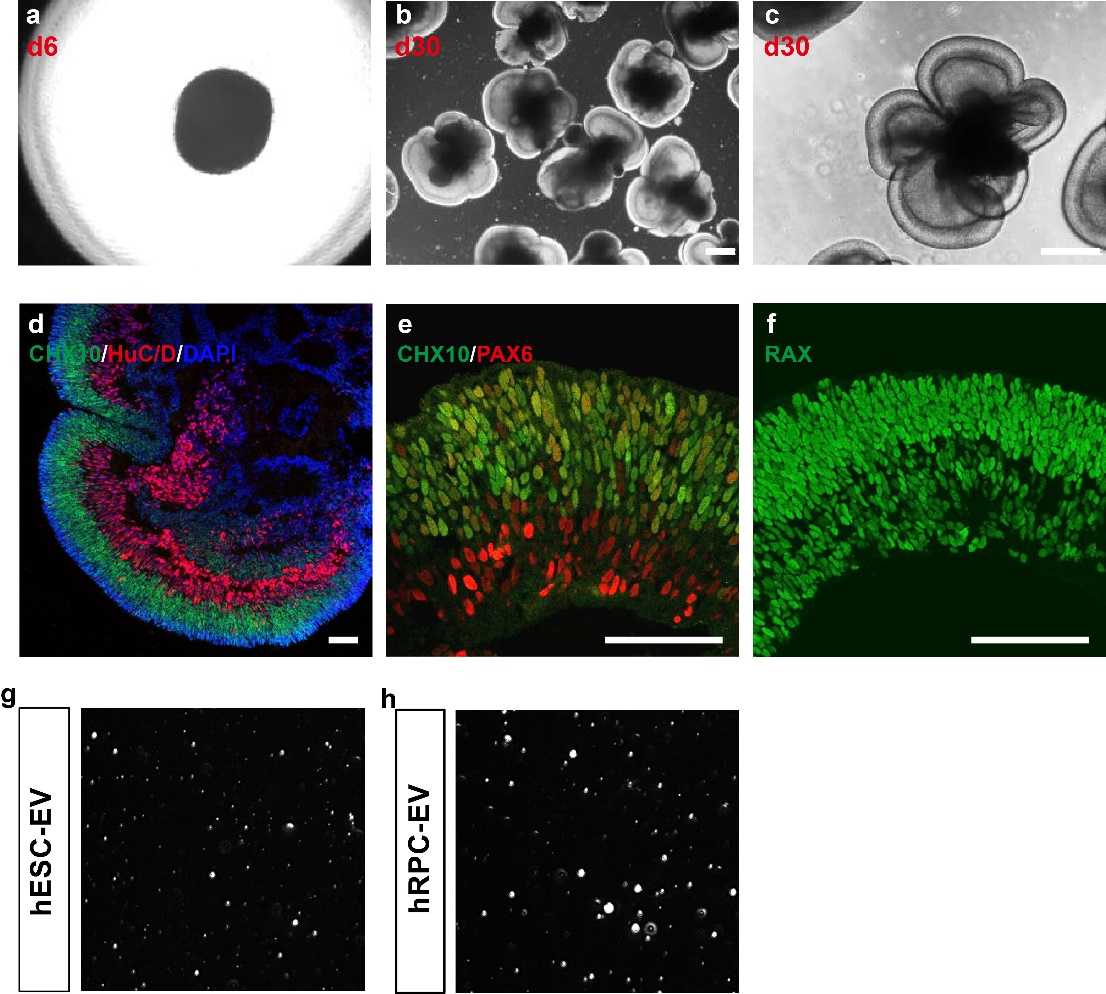


**Fig S1.** The induction and characterization of human retinal organoids. **a-c** Representative images of human embryonic stem cell-derived retinal organoids in different developmental stages (d6 and d30). **d-f** The expression of CHX10, HUCD, PAX6 and RAX in hEROs at d30. **g, h** Representative images of nanoparticle tracking analysis imaging for hESC-EVs and hRPC-EVs. Scale bar, 100 μm.


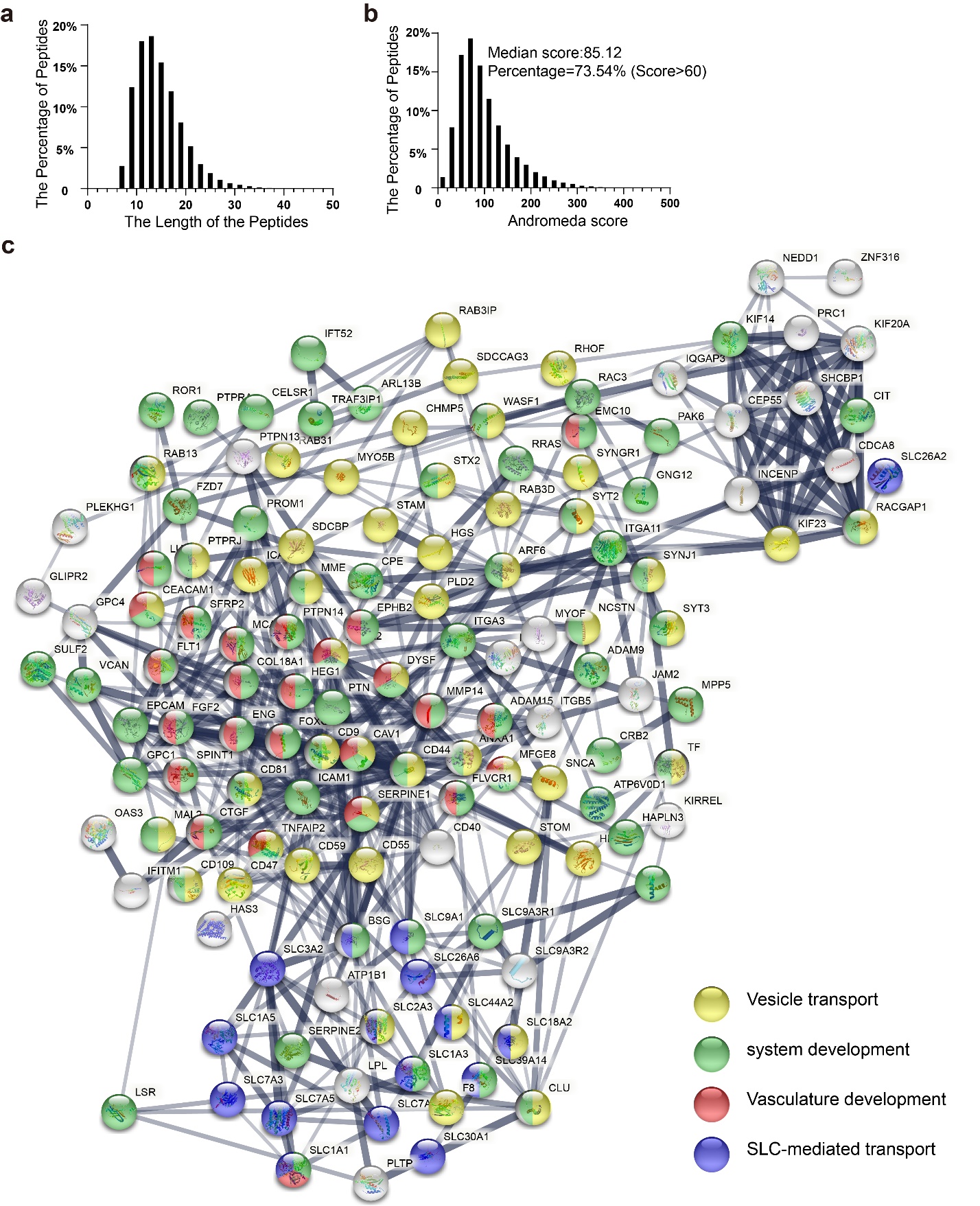


**Fig S2.** The quality control for LC–MS/MS and protein-protein interaction network. **a** The identified peptide length distribution in proteome analysis. **b** The identified peptide andromeda score in proteome analysis. **c** Protein-protein interaction network of proteins highly expressed in hESC-EVs compared to hESCs.


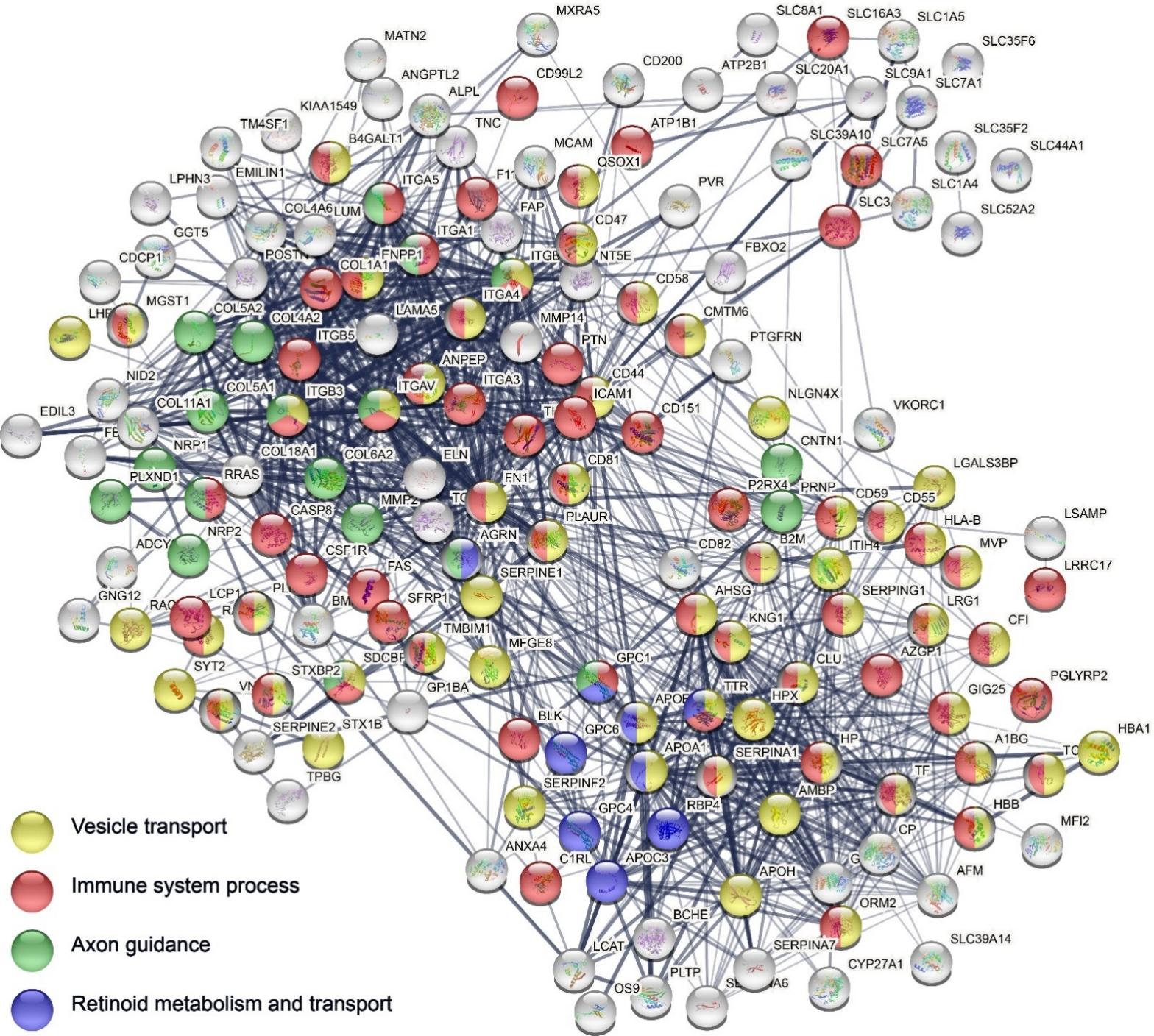


**Fig S3.** Protein-protein interaction network of proteins that highly expressed in hRPC-EVs compared to hRPCs.


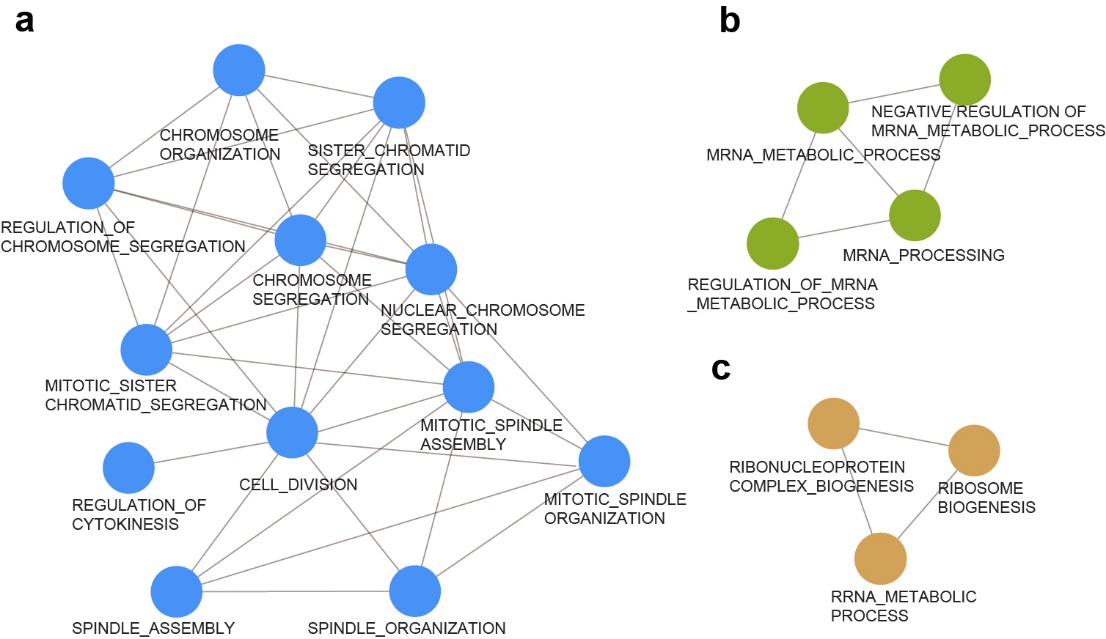


**Fig S4.** The networks of pathways enriched in hESC-EVs compared to hRPC-EVs. **a** The network of pathways related to cell division and chromosome segregation. **b** The network of pathways related to mRNA metabolic process. **c** The network of pathways related to ribosome.


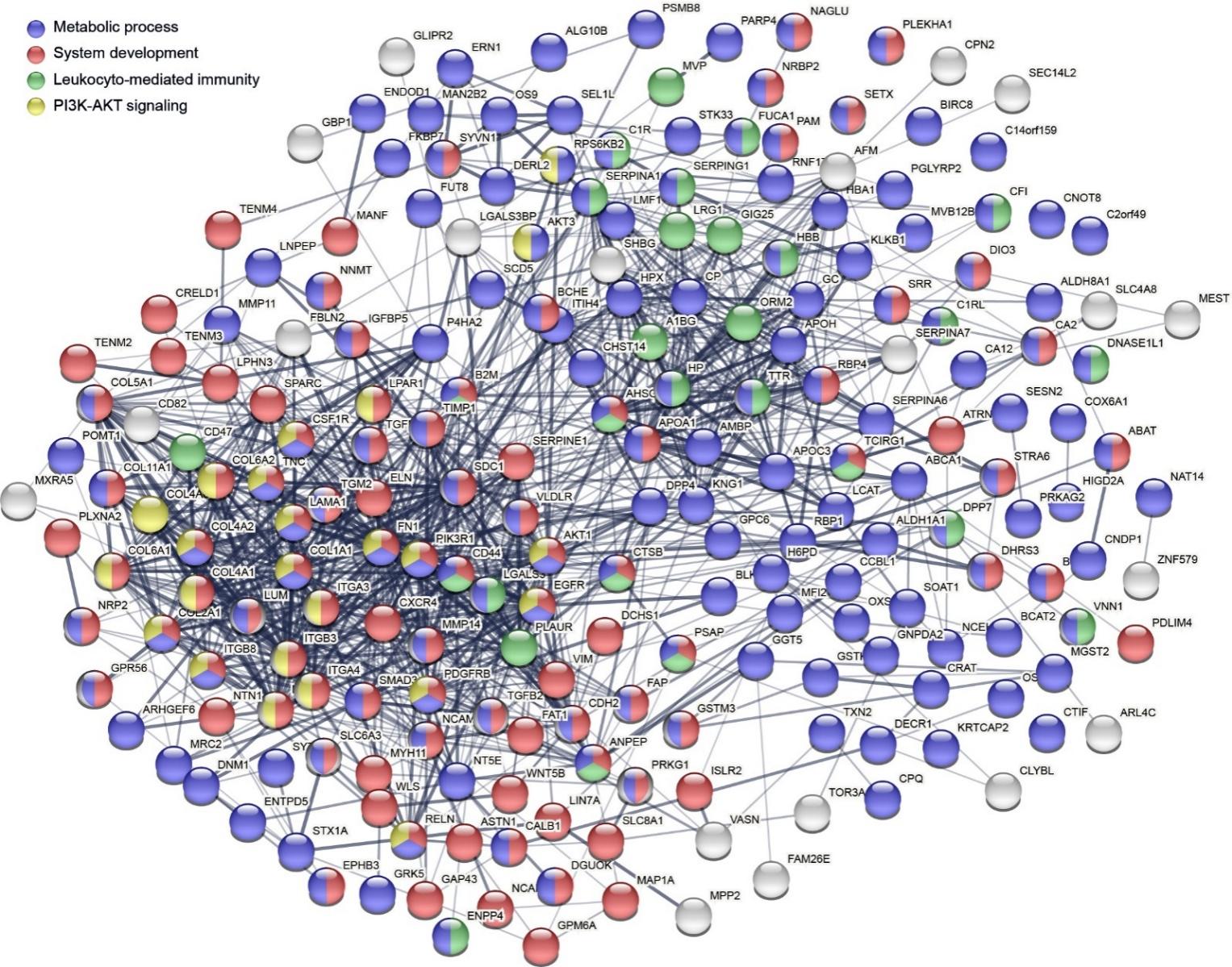


**Fig S5.** Protein-protein interaction network of proteins highly expressed in hRPC-EVs compared to hESC-EVs.


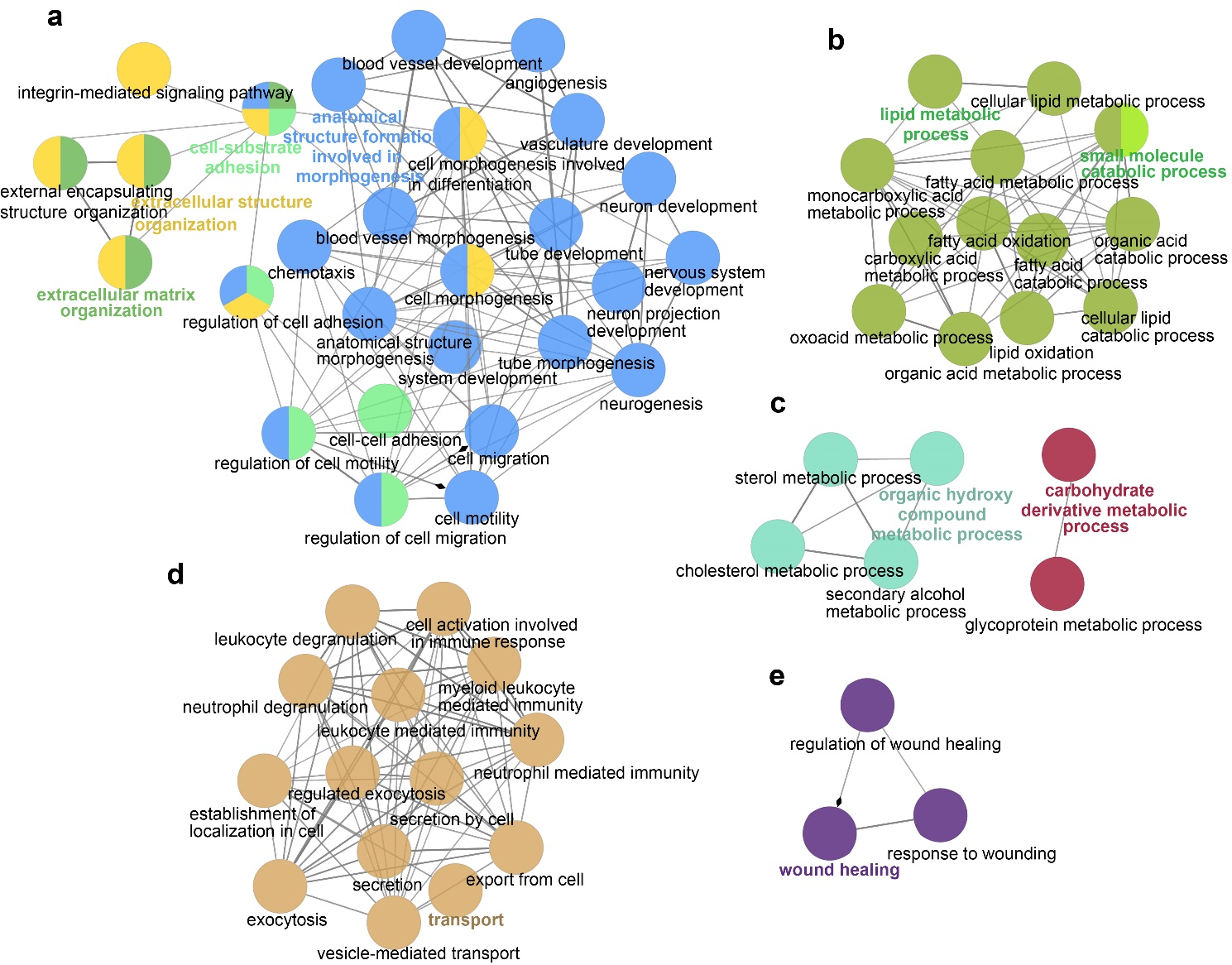


**Fig S6.** The networks of pathways enriched in hRPC-EVs compared with hESC-EVs. **a** The networks of ECM mediated signaling pathways, system and nervous development related pathways. **b** The networks of lipid and fatty acid metabolism related pathways. **c** The networks of sterols and glucose metabolism related pathways. **d** The networks of immune modulation related pathways. **e** Pathways of wounding healing.


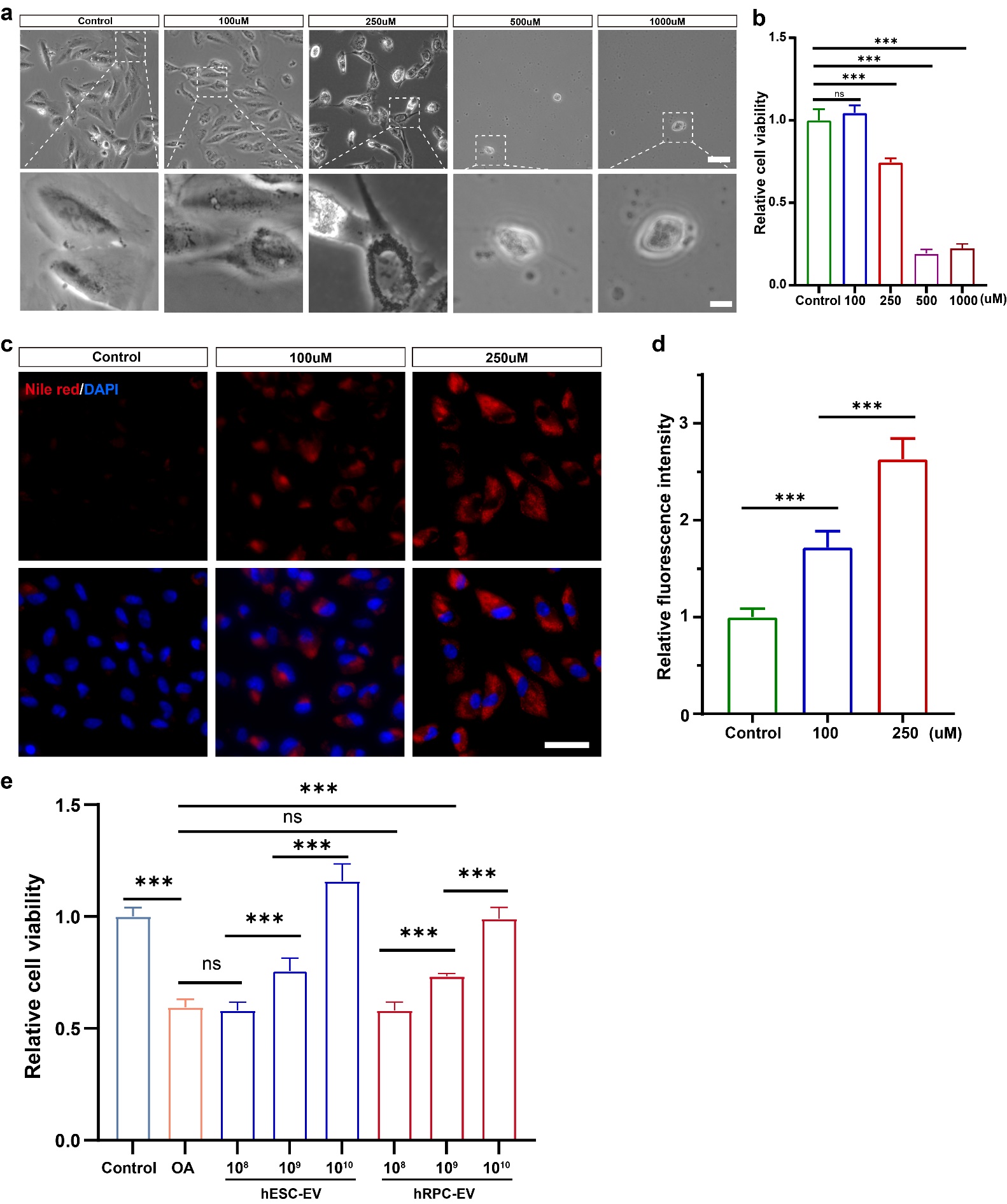


**Fig S7**. The establishment of lipotoxicity model in ARPE-19 cells and EVs treatment. **a, b** Representative images (a) and cell viability (b) of ARPE-19 cells after treatment with different dosages oleic acid for 24h. **c, d** Representative images of nile red staining and relative fluorescence intensity analysis at 24h. **e** The relative cell viability of ARPE-19 cells treated with different dosages of EVs as indicated. n = 6. Ns, not significant; ***P < 0.001 (one-way ANOVA for b, d, e). Scale bars: 50 μm (a, c), 10 μm (enlarged image of a).


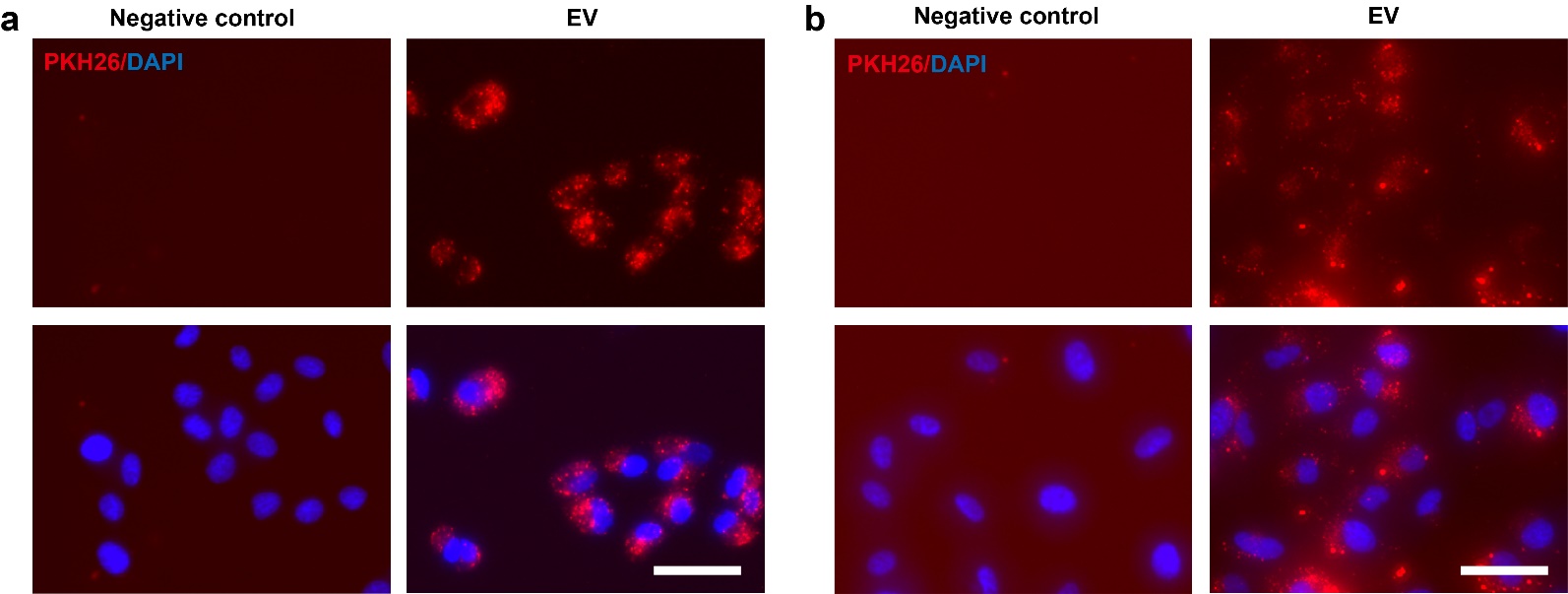


**Figure S8.** PKH26 labeling in ARPE-19 cells of negative control and EV groups. **a** Representative images of PKH26 signal in ARPE-19 cells of the negative control and EV group after 8 h coculture. **b** Representative images of PKH26 signal in ARPE-19 cells of the negative control and EV groups after 24 h coculture. Scale, 50 μm.


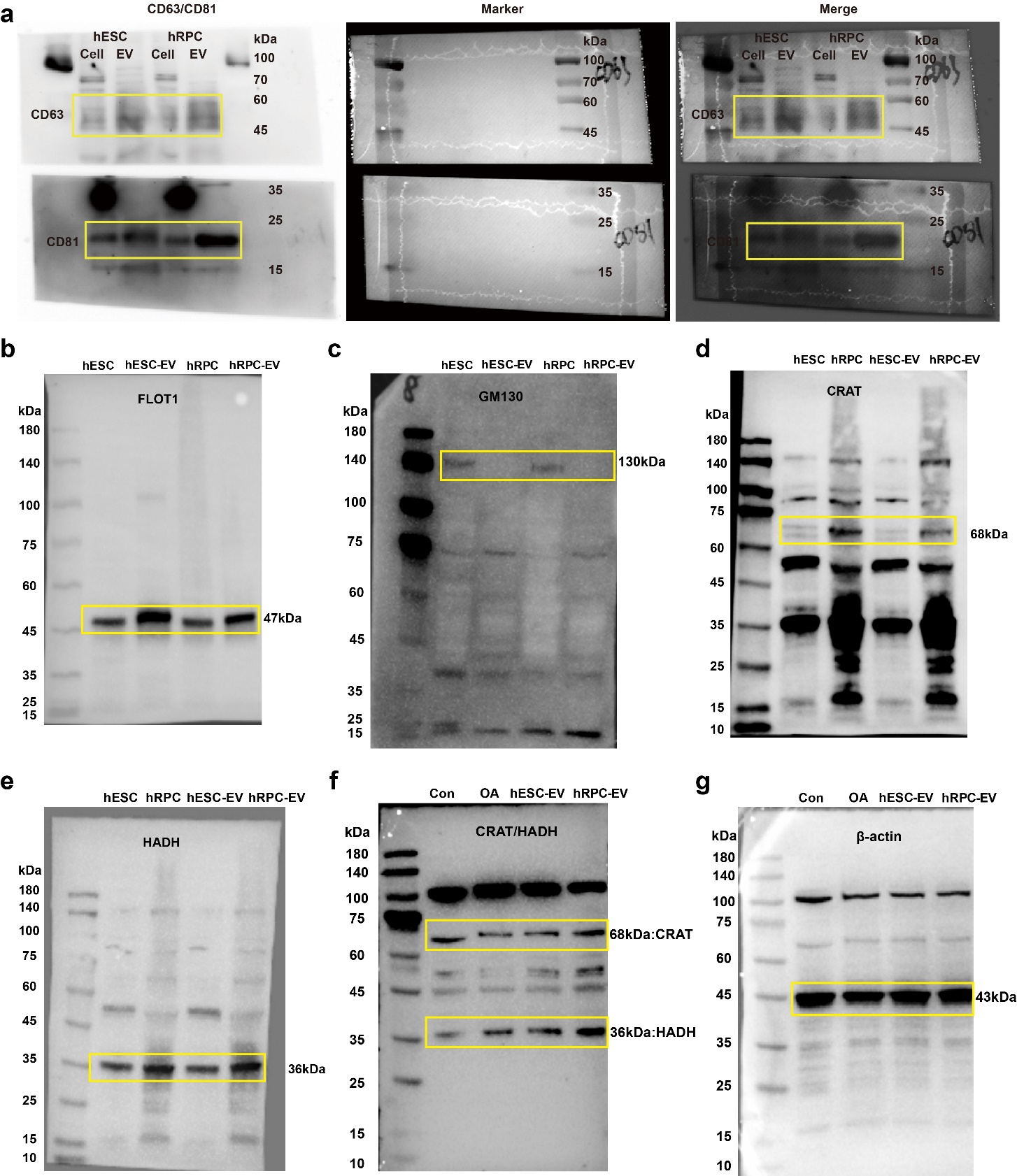


**Fig S9**. The whole western blot images. a, b, and c are affiliated to Fig 1g. d and e are affiliated to Fig 5k. f and g are affiliated to Fig 7e
